# Underlying Physics of Conductive Polymer Composites and Force Sensing Resistors (FSRs) under Static Loading Conditions

**DOI:** 10.3390/s17092108

**Published:** 2017-09-14

**Authors:** Leonel Paredes-Madrid, Carlos A. Palacio, Arnaldo Matute, Carlos A. Parra Vargas

**Affiliations:** 1 Faculty of Electronic and Biomedical Engineering, Universidad Antonio Nariño, Tunja 150001, Colombia; arnaldo.matute@uan.edu.co; 2 Faculty of Sciences, Universidad Antonio Nariño, Tunja 150001, Colombia; carlospalacio@uan.edu.co; 3 Grupo de Física de Materiales (GFM), Universidad Pedagógica y Tecnológica de Colombia, Tunja 150003, Colombia; carlos.parra@uptc.edu.co

**Keywords:** conductive polymer composite, FSR, piezoresistive sensor, quantum tunneling, force sensor, pressure sensor, quantum point contact

## Abstract

Conductive polymer composites are manufactured by randomly dispersing conductive particles along an insulating polymer matrix. Several authors have attempted to model the piezoresistive response of conductive polymer composites. However, all the proposed models rely upon experimental measurements of the electrical resistance at rest state. Similarly, the models available in literature assume a voltage-independent resistance and a stress-independent area for tunneling conduction. With the aim of developing and validating a more comprehensive model, a test bench capable of exerting controlled forces has been developed. Commercially available sensors—which are manufactured from conductive polymer composites—have been tested at different voltages and stresses, and a model has been derived on the basis of equations for the quantum tunneling conduction through thin insulating film layers. The resistance contribution from the contact resistance has been included in the model together with the resistance contribution from the conductive particles. The proposed model embraces a voltage-dependent behavior for the composite resistance, and a stress-dependent behavior for the tunneling conduction area. The proposed model is capable of predicting sensor current based upon information from the sourcing voltage and the applied stress. This study uses a physical (non-phenomenological) approach for all the phenomena discussed here.

## 1. Introduction

**Figure 1:**
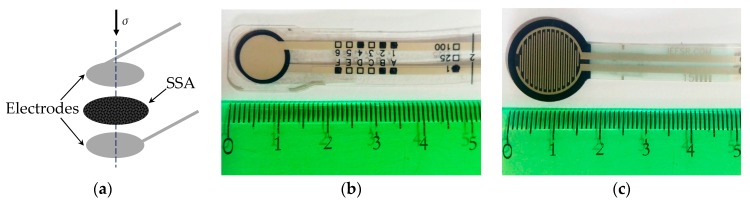
Schematic and pictures of commercial Force Sensing Resistors (FSRs). (**a**) Sketch of an FSR assembled under the Traditional Sandwich Element (TSE). The direction of normal stress (*σ*) is signaled with an arrow; (**b**) Picture of a FlexiForce A201-1 sensor; (**c**) Picture of an Interlink FSR 402 sensor. A centimeter ruler has been added for scaling purposes.

A Force Sensing Resistor (FSR) is a two-terminal passive device which exhibits a dramatic decrease in its electrical resistance when stress is exerted over the Sensor Sensitive Area (SSA). The SSA is usually made from conductive particles which are randomly dispersed in a non-conductive polymer resulting in a polymer composite; the SSA is later sandwiched between two metal electrodes to allow electrical measurements; the resulting device is an FSR as shown on the sketch of Figure 1a and on the picture of Figure 1b. This manufacturing process holds the designation of Traditional Sandwich Element (TSE). It is also possible to assemble an FSR using non-aligned electrodes (NAEE) or transverse electrodes (TEP) as the sensor on Figure 1c. Wang et al. [1,2] have demonstrated that the location of the electrodes notably influences the creep behavior of FSRs. 

The characterization of materials that can be used in the SSA manufacturing is currently an active research area. A broad range of materials have been evaluated as the non-conductive element in the fabrication of SSA, but elastomers, rubbers and polydimethylsilicone (PDMS) are the preferably chosen polymers as reported by Stassi et al. [3]. Likewise, conductive particles can be obtained from different metals, such as Nickel or Cooper, with particles sizes within the range of tens of nanometers up to a few micrometers [4,5]. Carbon black and carbon nanotubes have also been implemented as conductive phases in the insulating matrix [6,7].

As can be noticed from the previously cited studies, the research possibilities are vast within the manufacturing of SSA since they comprise multiple design parameters, such as: electrode configuration (TSE, NAEE, TEP and hybrid topologies), type of insulating material (elastomer, rubber, resins, metal-oxides and thermostable polymers), and properties of the conductive particles (material and size of particles, mass ratio of the conductive particles to the insulating material). Therefore, multiple designations have been given in literature to these kinds of smart materials, but the key aspect to categorize them has been placed on the type of insulating element. Hence, the most common designations that can be found in regard to the SSA are: conductive polymer [8], semiconductive polymer [9], and rubber/elastomer/PDMS composite with conductive nanoparticles [10]. Likewise, the phenomenological designation of piezoresistive sensor is also common in literature [11]. Henceforth in this article, the designation of polymer composite is used when discussing about the material employed in the manufacturing of the SSA.

On the other hand, the designation of FSR has been preferably used to refer to off-the-shelf sensors which can be readily integrated into a given application. Several models of FSRs are currently commercially available [12], but the most-widely used brands of FSRs are the family of FlexiForce A201/A401 sensors, manufactured by Tekscan, Inc. (Boston, MA, USA) [13,14,15] and the family of FSR 40X sensors manufactured by Interlink Electronics, Inc. (Westlake Village, CA, USA) [16,17,18]. Pictures of both sensors are shown in Figure 1b,c, respectively. Both manufacturers offer a broad range of force-sensing solutions in terms of nominal force ranges, sensor dimensions, interface circuits and end-user software, such as the popular F-scan in-shoe analysis system which is intended for gait analysis [19].

It must be recalled that the designation of thin insulating film/layer has been used in literature to describe and model the conduction mechanisms of polymer composites [20,21]. A polymer composite operates on the basis of either quantum tunneling or percolation; although both phenomena can actually occur simultaneously [3]. When subjected to stress, the average inter-particle separation of the polymer composite changes, and consequently, the electrical resistance of the SSA varies. In the 1960s, Simmons [21] provided the foundation physics for the quantum tunneling conduction of thin insulating layers. His model accurately predicted the current-voltage (*I-U*) relationship that Fisher and Giaever [20] have previously reported when characterizing thin insulating film layers fabricated from aluminum oxide. Later models have been developed based upon the original formulation from Simmons [21]; such models are more complex as they embrace additional parameters such as: particle size on the conductive filler [9], creep response of the polymer composite [22] and mass ratio of the conductive phase to the insulating matrix [23].

The afore-cited models of Kalantari et al. [9], Zhang et al. [22] and Wang et al. [23] share one thing in common; they describe the relative variation of the electrical resistance when the polymer composite is subjected to stress. The relative variation of resistance is represented in regard to one of the following magnitudes: the original resistance immediately upon the application of stress [9,22], or the original resistance when the polymer composite is unloaded [23]. In practice, this implies that the aforementioned models rely upon experimental readings to describe the *I-U*, stress-resistance (*σ-R*), and the time-resistance (*t-R*) relationships. Nonetheless, the original formulation from Simmons [21] does not comprise any type of experimental readings. Instead, the model from Simmons [21] is based upon physical constants, e.g., electron mass and charge, Planck constant, and upon different properties from the conductive and the non-conductive elements, e.g., average inter-particle separation and work function of each element.

The main contribution of this article is to derive a theoretical model capable of predicting the absolute *I-U* and *σ-R* relationships of polymer composites and FSRs when subjected to stress. The proposed model has mainly two differences with the aforesaid studies from Kalantari et al. [9], Zhang et al. [22] and Wang et al. [23] as next mentioned: first, as the proposed model describes the absolute *I-U* and *σ-R* relationships, it does not require experimental measurements of the electrical resistance; this is important because the proposed model is capable of making predictions relying solely on simulation, and second, the proposed model embraces two additional parameters that the above mentioned studies have ignored or have assumed as constant; these are: the stress-dependent behavior of the area (*A*) for quantum tunneling conduction, and the influence of the input voltage (*U*) over the electrical resistance.

Finally, it must be recalled that FSRs are made from a polymeric binder, and thus, a rheological behavior is observed in the electrical resistance of the device [24]. The rheological response of polymers yields inaccuracies that fall within the following types of errors: hysteresis [25], creep [26], and loss of sensitivity under continuous operation [27,28]. In this article, and for the sake of paper length, the rheological behavior of polymer composites is not taken into account which is a limitation of the proposed model. Similarly, this work is based upon the Simmons model [21] for the current conduction through thin insulating layers which was derived on the basis of two approximations: the WKB and approximated evaluation of integrals, and thereby, the experimental data do not perfectly fit the proposed model.

The rest of this paper is organized as follows: the conduction mechanisms of polymer composites and FSRs are described in Section 2 with special emphasis on the quantum tunneling effect and the contact resistance. In Section 3, a review is presented for the models that predict the relative variation of the *I-U* and *σ-R* relationships of polymer composites. In Section 4, the derivation of the proposed model is addressed, followed in Section 5 by a presentation of the experimental results, and conclusions on Section 6. Two appendixes have been included in this article: Appendix A presents a review on the concepts of current density (*J*) electrical current (*I*), and electrical resistance per unit area (*R_A_*). A full understanding of such concepts is required to comprehend Simmons’ model [21]. Appendix B describes the steps followed by Simmons to derive his model.

The tests conducted in this study were performed over A201-1 and FSR 402 sensors, manufactured by Tekscan, Inc. (South Boston, MA, USA) and Interlink Electronics, Inc. (Camarillo, CA, USA), respectively. The nominal range of both sensors is 4.5 N and 20 N, respectively.

## 2. Conduction Mechanism of Polymer Composites and Contact Resistance of FSRs 

The predominant conduction mechanisms of polymer composites and FSRs are quantum tunneling and percolation. This has been stated by Stassi et al. [3], but it is also true for any type of thin insulating film as experimentally demonstrated by Fisher and Giaever [20], and later modeled by Simmons [21]. A description of each phenomenon is given next, but more emphasis is placed on quantum tunneling since it describes the conduction mechanism of the A201-1 and the FSR 402 sensors. Readers may refer to Hou et al. [29], Knite et al. [30], and Chen et al. [31] for further details on percolation.

### 2.1. Quantum Tunneling as Conduction Mechanism

**Figure 2:**
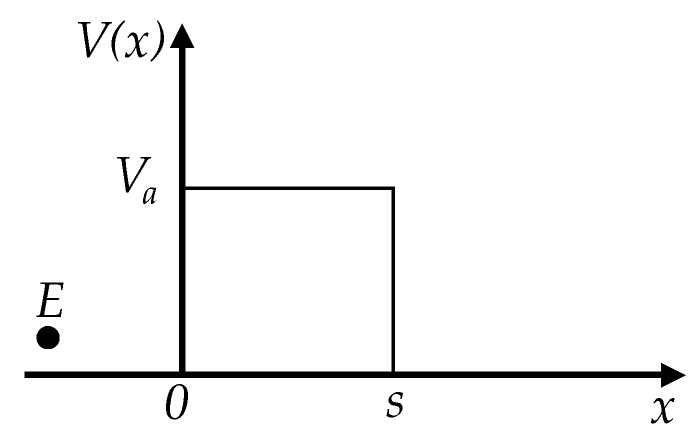
Rectangular potential barrier with height *V_a_* and width *s* next to a particle with energy *E*.

In quantum mechanics, the tunnel effect occurs when a particle crosses a potential barrier of height *V*(*x*) and width *s* as shown in Figure 2. The solution for this problem can be found from the time-independent Schrödinger equation for the wave function *ψ*(*x*):

(1) $$H\psi (x)=\left[-\frac{\hbar^{2}}{2m}\frac{d^{2}}{dx^{2}}+V(x)\right]\psi (x)=E\psi (x)$$

where *m* and *E* are the mass and energy of the particle, respectively. *H* is the Hamiltonian and *ħ* is the Planck constant (*h*) divided by 2π. In the simplest form, the potential barrier *V*(*x*) has the form of a rectangle with height *V_a_* and width *s*. Hence, the potential barrier *V*(*x*) can be represented as a piecewise function:

(2) $$V(x)=\{\begin{array}{ll} 0 & if\;x<0\\ V_{a} & if\;0<x<s\\ 0 & if\;x>s \end{array}$$

Solving Equation (1) for the rectangular barrier of Equation (2) implies finding the probability for the incident particle with energy *E* to cross the potential barrier *V*(*x*). This is a well-studied phenomenon in quantum mechanics [32]. The solution is also a piecewise function in terms of the probability of particle transmission (*T*) with the three possible energy states of *E*, that is: *E* < *V_a_*, *E* = *V_a_* and *E* > *V_a_*. Solutions for each case are next presented:

If *E < V_a_*:

(3) $$T=\frac{1}{1+\frac{V_{a}^{2}\sinh(k_{1}s)}{4E(V_{a}-E)}}$$

If *E = V_a_*

(4) $$T=\frac{1}{1+ms^{2}V_{a}/2\hbar^{2}}$$

If *E > V_a_*

(5) $$T=\frac{1}{1+\frac{V_{a}^{2}\sin^{2}(k_{2}s)}{4E(E-V_{a})}}$$

The arguments of the sine and hyperbolic sine have been conveniently stated in terms of *k*_1_, *k*_2_:

$$k_{1}=\sqrt{2m(V_{a}-E)/\hbar^{2}}$$

$$k_{2}=\sqrt{2m(E-V_{a})/\hbar^{2}}$$

**Figure 3:**
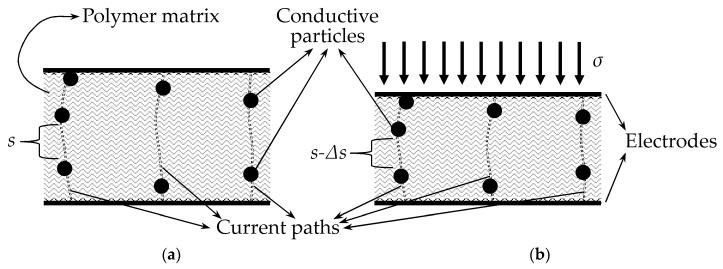
Sketch of a polymer composite with randomly spaced conductive particles and multiple current paths. (**a**) Unloaded polymer composite depicting a tunneling path of width *s*; (**b**) Polymer composite under applied stress (*σ*) depicting a shortening in the tunneling path from *s* down to *s-Δs*. The applied stress is exerted over the sensor electrodes causing compression in the polymer matrix.

An FSR operates on the basis of quantum tunneling, with multiple paths for particle transmission (current paths) as shown in Figure 3. The transmitted particles are electrons, and the polymer matrix acts as the potential energy barrier. The role of the conductive nanoparticles is to allow electron bridging between consecutive barriers. The model sketched on Figure 3a has been widely used by authors to describe the conduction mechanism of polymer composites [7,9,22], but to the authors’ knowledge it was initially proposed by Fisher and Giaever [20].

When mechanical stress (*σ*) is applied across the sensor, most of the tunneling paths shrink; this phenomenon occurs when particle concentration is below the percolation threshold. Figure 3b depicts a tunneling path subjected to stress that shortens from *s* down to *s-Δs*, and thus, a larger flow of electrons is observed as theoretically predicted by Equations (3)–(5). An increase in the number of current paths is actually observed as *σ* grows, but this condition has been deliberately not shown in Figure 3 for the sake of simplicity. This issue is later discussed in the upcoming sections.

Unfortunately, Equations (3)–(5) are not straightforward usable in practice, because they are stated in terms of the particle energy, *E*, that is a function of: the applied voltage across the thin insulating film, *U*, and the kinetic energy that follows a Fermi-Dirac probability distribution. Nonetheless, Lantada et al. [10] developed a model based on Equation (3) that is capable of predicting the relative variation of resistance in elastomeric composites. The model from Lantada et al. is not addressed in this article since its formulation is similar to those from Kalantari et al. [9], Zhang et al. [22] and Wang et al. [23].

A different approach was developed by Simmons [21] to model the *J-U* relationship in thin insulating layers. In his study, a theoretical model was developed on the basis of the applied voltage, *U*, and current density, *J*, which are electrical variables much easier to measure [21]. The equations obtained by Simmons are next presented, but readers may refer to the Appendix B for further details on his theoretical derivation.

### 2.2. Current Density-Voltage (J-U) Relationship of Thin Insulating Film Layers. A Review on the Theoretical Work from Simmons [21]

For the sake of simplicity, a single potential barrier is henceforth considered to study the variations in the current density, *J*, and the total current, *I*. This is a reasonable approach based on the fact that the net current flowing through a polymer composite is the sum of the individual contributions from all the quantum-tunneling bridges, see Figure 3. This consideration has been made by several authors to describe the *I-U* relationship of polymer composites [6,7,9,22,23]. The quantum-tunneling bridges from Figure 3 operate as individual thin insulating film layers, and the net contribution can be calculated from the equations derived by Simmons [21].

The variations in the current density are produced by a combination of changes at the barrier width, *s*, applied voltage, *U*, and the height of the rectangular potential barrier, *V_a_*. In this article, only the rectangular potential barrier is considered. However, the more general case of image potential has been also considered by Simmons [21]. 

The theoretical model of Simmons was obtained on the basis of the WKB approximation. The equations are piecewise functions in terms of *U* in regard with *V_a_* divided by the electron charge, *e*. The equations are presented below:

If *U* ≈ 0

(6) $$J(U,s)=\frac{3\sqrt{2mV_{a}}}{2s}{\left(\frac{e}{h}\right)}^{2}U\exp\left(-\frac{4\pi s}{h}\sqrt{2mV_{a}}\right)$$

If *U* < *V_a_*/*e*

(7) $$\begin{array}{l} J(U,s)=\left(\frac{e}{2\pi hs^{2}}\right)\{\left(V_{a}-\frac{eU}{2}\right)\exp\left[-\frac{4\pi s}{h}\sqrt{2m\left(V_{a}-\frac{eU}{2}\right)}\right]\\ \;\;\;\;\;\;\;\;\;\;\;\;\;\;\;\;\;\;\;\;\;-\left(V_{a}+\frac{eU}{2}\right)\exp\left[-\frac{4\pi s}{h}\sqrt{2m\left(V_{a}+\frac{eU}{2}\right)}\right]\} \end{array}$$

If *U* > *V_a_*/*e*

(8) $$\begin{array}{l} J(U,s)=\frac{2.2e^{3}U^{2}}{8\pi hV_{a}s^{2}}\{\exp\left[-\frac{8\pi s}{2.96heU}\sqrt{2mV_{a}^{3}}\right]\\ -\left(1+\frac{2eU}{V_{a}}\right)\exp\left[-\frac{8\pi s}{2.96heU}\sqrt{2mV_{a}^{3}\left(1+\frac{2eU}{V_{a}}\right)}\right]\} \end{array}$$

Following the explanation from previous sections, the barrier shrinks from *s* down to *s-Δs*, when subjected to stress, see Figure 3. Hence, a larger flow of electrons is predicted regardless of the magnitude of *U*, i.e., Equations (6)–(8) predict an increase in *J* when *s* is reduced. Similarly, if *U* is increased at constant stress, a larger value of *J* is expected.

Besides the WKB approximation, Simmons employed approximations when solving the integrals that yielded the previously stated equations. The errors associated with such approximations are typically below 1% with a maximum error of 6% for Equation (7); such error margins are acceptable but have the consequence of producing discontinuous outputs around *U* ≈ 0 and around *U* = *V_a_*/*e*. Further detail on this subject is presented in Appendix B.

**Figure 4:**
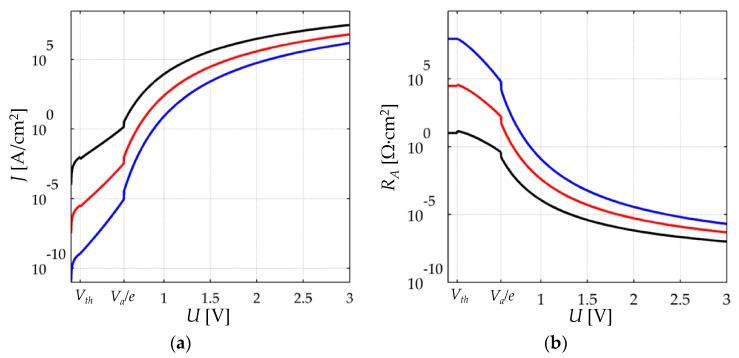
Simulated data for a thin insulating film as obtained from the Simmons’ theoretical equations [21] for *V_a_* = 0.57 eV, *s* = 6 nm (blue), *s* = 5 nm (red) and *s* = 4 nm (black). Incremental forces/stresses yield lower values of *s*. (**a**) Current density (*J*) as a function of the applied voltage (*U*); (**b**) Resistance per unit area (*R_A_*) as a function of *U*.

Based upon Equations (6)–(8), simulated data are presented in Figure 4a for *s* = 6 nm, *s* = 5 nm and *s* = 4 nm. Lower *s* values are stemmed from large applied forces/stresses as later discussed in Section 3. The plots of Figure 4 were obtained assuming a constant *V_a_* = 0.57 eV. These are typical values for *V_a_* and *s* according to previous experimental results from Zhang [22], and Fisher and Giaever [20]. With the aim of avoiding confusion in the ambiguous definition of *U* ≈ 0 in Equation (6); the concept of the millivolt threshold (*V*th) is introduced by the authors. *V*th is defined as the threshold voltage at which the polymer composite no longer exhibits an ohmic response. *V*th is a quantity that varies from one material to another, just as *V_a_* does. However, in practice *V*th < *V_a_/e* as it defines the transition between Equations (6) and (7). Henceforth in this article, the piecewise Equations (6)–(8) are redefined as:

If *U* < *V*th
  Equation (6) models the behavior of the polymer composite.
If *V*th < *U* < *V_a_/e*
  Equation (7) models the behavior of the polymer composite.
If *U > V_a_/e*
  Equation (8) models the behavior of the polymer composite.

A distinction is here introduced between polymer composites and FSRs. As previously stated, when a polymer composite is sandwiched between metal electrodes an FSR is obtained. In practice, Equations (6)–(8) model the current density conduction of polymer composites, but in order to use them with FSRs, the resistance contribution from the contact resistance must be considered. Upcoming sections discuss about this condition. 

In Figure 4a, *V*th has been set arbitrary to 100 mV for simulation purposes, but *V*th can be experimentally measured for a given material. Note the discontinuous output around *U* = *V*th and around *U* = *V_a_*/*e* caused by the aforesaid approximations. 

The electrical resistance per unit area (*R_A_*) is defined as the quotient between *U* and *J*, see Equation (A4) in Appendix A for further details on *R_A_*. The electrical resistance per unit area, *R_A_*, is plotted on Figure 4b by using the same data from Figure 4a.

Note from the plot of Figure 4b, that *R_A_* exhibits a voltage-dependent behavior, with *R_A_* decreasing as *U* grows. Only when *U < V*th, the value of *R_A_* can be assumed as voltage-independent. The simulation plot of Figure 4b is in contradiction with the models that describe the relative variation of the electrical resistance, that is, the models of Kalantari et al. [9], Zhang et al. [22] and Wang et al. [23]. In such studies, it is assumed that the electrical resistance of a polymer composite (*R_Pol_*) is a voltage-independent quantity that can be computed from:

(9) $$R_{Pol}=\frac{2s}{3A\sqrt{2mV_{a}}}{\left(\frac{h}{e}\right)}^{2}\exp\left(\frac{4\pi s}{h}\sqrt{2mV_{a}}\right)$$

where *R_Pol_* is obtained by a combination of Equation (6), Equation (A2) and the Ohm’s law, with *A* representing the effective area for the electrons to cross the potential barrier, i.e., the tunneling conduction area. However, Equation (6) is only valid in the millivolt range, and thus, Equation (9) is only applicable when *U* < *V*th; but still, the aforesaid authors have employed Equation (9) when fitting experimental measurements with *U* equal to 5 V [9], or when performing experimental measurements of resistance with digital multimeters [22,23,33]. This inconsistency is the main motivation behind the proposal of a new model for polymer composites as later addressed in Section 4. Experimental results from Section 5 support the Simmons’ prediction from Figure 4b in which *R_A_* exhibits a voltage-dependent behavior.

Readers may refer to Appendix A for a thorough review on the relationship between *J* and *I.* On this topic, it must be recalled that the cross-section area *A* is the effective area for the electrons to cross the potential barrier, and so, it must not be confused with the sensor physical area (*A_FSR_*). Finally, it must be pointed out that some authors refer to *R_Pol_* on Equation (9) as the tunneling resistance [1,34]. The designations of polymer composite resistance and tunneling resistance are indistinctly used in this article, but in Section 4, new expressions for *R_Pol_* are introduced.

### 2.3. Contact Resistance in FSRs

**Figure 5:**
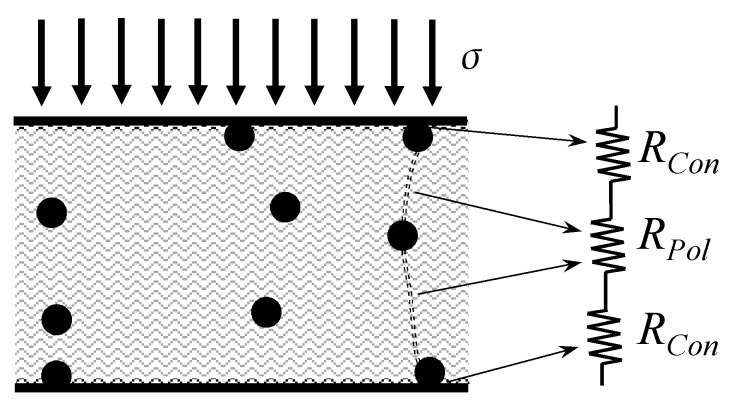
Schematic view of an FSR representing the contact resistance (*R_Con_*) and the resistance of the polymer composite (*R_Pol_*) as in Equation (9). Direction of applied stress (*σ*) is signaled with arrows.

To the authors’ knowledge, the proposal of contact resistance in FSRs (*R_con_*) was initially introduced by Ruschau et al. [35], but Kalantari et al. [9] further developed the concept, as an analytic expression for *R_Pol_* was included together with *R_Con_*. In their work, an FSR was manufactured using Linqstat polymer and carbon black as conductive phase. Kalantari et al. proposed that the resistance of an FSR can be decomposed as shown in Figure 5:

Finally, the total resistance (*R_total_*) can be written as:

(10) $$R_{total}=2R_{Con}+R_{Pol}$$

A theoretical model for *R_Con_* can be found on the basis of the following expression [9]:

(11) $$R_{Con}(F)=\frac{\rho_{1}+\rho_{2}}{4}\sqrt{\frac{\pi H}{F}}$$

where *ρ*_1_ and *ρ*_2_ are the resistivity of the materials in contact, *H* is the Meyer hardness of the softer member and *F* is the applied force. The usage of the applied force, *F*, as the input of Equation (11) was done to keep the original formulation from Kalantari et al. [9]. In Section 4.2, an expression for the contact resistance is introduced using the applied stress, *σ*, as input.

**Figure 6:**
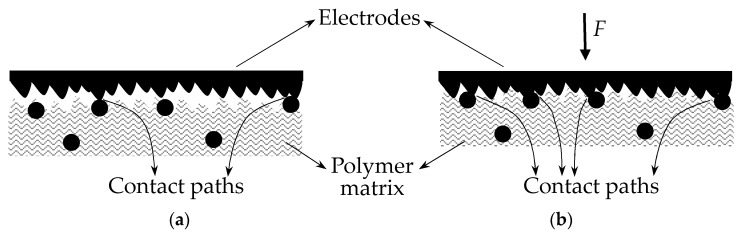
Schematic view at a microscopic scale of the contact paths at different loading conditions. Conductive particles are represented as black points. (**a**) Paths at sensor rest, *F = 0*; (**b**) Paths during loading condition *F > 0.*

A physical explanation for *R_Con_* can be found by recalling the roughness of surfaces at a microscopic scale. The contact resistance takes place on the asperities occurring between the sensor electrodes and the conductive particles; this explanation has been proposed by Kalantari et al. [9]; so a similar sketch of their proposal is here reproduced on Figure 6. The underlying basis for the contact resistance can be found on the plastic and elastic interactions occurring between the conductive particles and the sensor electrodes at a microscopic level; such description has been proposed by Ruschau et al. [35], and later modeled by Mikrajuddin et al. [36].

Note from Equation (11), that the contact resistance, *R_Con_*, is reduced in an inverse square-root of *F*. This is a quite reasonable model based on the representation from Figure 6 that depicts more current paths when force is applied. However, Kalantari et al. did not considered in their model that as the applied force increases, the area *A* is also increased, i.e., more current paths are formed, and thus, the effective area for tunneling conduction is also increased.

In the work of Kalantari et al. [9], the assumption of constant area is evident from Equations (3)–(7), because it is assumed that only strain is affected with force, readers may refer to Equation (6) at [9]. In other theoretical models of polymer composites, the effective area *A* is also assumed as a force-independent quantity [22,23]. However, this assumption is demonstrated to be inappropriate, at least for the A201-1 and FSR 402 sensors. Experimental results shown in Section 5 demonstrate that *A* grows as the applied force is increased, and simultaneously, a reduction in *R_Con_* is also observed for incremental values of *F*.

### 2.4. Percolation Mechanism

A polymer composite working on the basis of percolation exhibits an increase in the total electrical resistance when subjected to stress. In contrast, a polymer composite working on the basis of quantum tunneling shows a resistance decrement when loading is applied as shown in Figure 4. Under percolation operation, such a behavior is observed because the polymer matrix has a particle concentration above the percolation threshold, and therefore, the particles touch each other when the sensor is at rest, that is, when the applied stress, *σ*, is equal to zero. However, when subjected to stress, the particles move apart from each other, and consequently, the resistance of the polymer composite increases. It must be highlighted that when the particle concentration is below the percolation threshold, the predominant conduction mechanism is quantum tunneling. However, both phenomena—percolation and quantum tunneling—actually occur simultaneously in a polymer composite [3,6].

## 3. A Review on the Models that Predict the *I-U* and *σ-R* Relationships of Polymer Composites

In this section, it is presented a brief description of the models that predict the relative variation of resistance in polymer composites. The models of Zhang et al. [22], Wang et al. [23] and Kalantari et al. [9] are individually addressed. The original notation from the authors has been kept for all symbols except for the parameters describing the sensor area. This was necessary for two reasons: the aforesaid authors do not employ a unique nomenclature, and second, a distinction was introduced in this article for the sensor physical area, *A_FSR_*, and the effective area for tunneling conduction, *A*. The start point for all the models is the following equation:

(12) $$R(\sigma )=\frac{L(R_{Pol}+R_{par})}{S}$$

where *R*(*σ*) is the composite resistance as a function of the applied stress, *L* is the number of particles forming one conductive path (series-connected), *S* is the total number of conductive paths, *R_Pol_* is given by Equation (9), and *R_par_* is the resistance of a single conductive particle. Zhang et al., Wang et al. and Kalantari et al. stated that *R_par_* is negligible when compared to the resistance of the polymer composite—the tunneling resistance *R_Pol_*—and thus, Equation (12) can be simplified to:

(13) $$R(\sigma )\approx \frac{LR_{Pol}}{S}$$

Let *R*_0_ be the resistance of the polymer composite at rest state, i.e., when *σ =* 0. In practice, *R*_0_ is experimentally measured with a multimeter before sensor testing [9,22,23]. By replacing Equation (9) at the Equation (13), and dividing by *R*_0_, the following expression can be obtained:

(14) $$\frac{R(\sigma )}{R_{0}}=\frac{s}{s_{0}}\exp\left((s-s_{0})\frac{4\pi }{h}\sqrt{2mV_{a}}\right)$$

where *s*_0_ is the inter-particle separation in the polymer composite at sensor rest and *s* is the inter-particle separation when subjected to stress. The quotient *L*/*S* has been removed from the Equation (14), because Zhang et al. and Kalantari et al. considered that such a quotient is force independent. Conversely, Wang et al. stated in their model that the number of conductive paths actually grows as the applied stress increases. The authors’ proposal on this topic is addressed on Section 4. Variations of Equation (14) have been developed by Zhang et al., Wang et al. and Kalantari et al. as next described:

### 3.1. The Model of Zhang et al. [22]

Zhang et al. were the first to propose a model for the relative variation of resistance in polymer composites. Later models from Kalantari et al. and Wang et al. are based on the original formulation of Zhang et al*.*, and hence, special emphasis is placed on describing the original formulation from Zhang et al., whereas the other two models are briefly described.

Previous attempts on modeling the piezoresistance of polymer composites have been carried out by Carmona et al. [37] and Ruschau et al. [35]. However, it must be remarked that the Carmona’s derivation was obtained on the basis of assuming classical percolation conduction through the polymer composite, whereas Ruschau et al. put more emphasis on the tunneling resistance at the contacts but a theoretical model for such phenomenon was not provided. Conversely, the model from Zhang et al. and their successors were obtained on the basis of quantum tunneling. Zhang et al. developed two models for the relative variation of resistance. The first model is focused on static forces whereas the second predicts the creep response of polymer composites. 

Given the compressive modulus (*M*) of the insulating polymer matrix subjected to stress, *σ*. The strain (ε) can be found from the quotient *ε = σ/M*. 

Given the inter-particle separation in the polymer composite at rest state, *s*_0_. An expression for *s*_0_ can be found from the particle diameter (*D*) and filler volume fraction (*θ*) as follows:

(15) $$s_{0}=D\left[{\left(\frac{\pi }{6\theta }\right)}^{1/3}-1\right]$$

The filler volume fraction can be understood as the density ratio of the conductive nanoparticles to the insulating polymer matrix, readers may refer to Equation (19) at [23] for further details on this topic. The relationship between the inter-particle separation, *s,* and strain, *ε*, can be expressed as:

(16) $$s=s_{0}(1-\varepsilon )=s_{0}(1-\sigma /M)$$

A combination of Equations (9), (15) and (16) yields the first model of Zhang et al. in regard to the electrical resistance at sensor rest, *R*_0_. The designation “in regard to *R*_0_” implies dividing the resulting equation by *R*_0_ as in Equation (14).

(17) $$\frac{R(\sigma )}{R_{0}}=\left(1-\frac{\sigma }{M}\right)\exp\left[-\frac{\sigma D}{M}\frac{4\pi }{h}\sqrt{2mV_{a}}\left[{\left(\frac{\pi }{6\theta }\right)}^{1/3}-1\right]\right]$$

Three important facts can be yielded from Equation (17); first note that it is only capable of modeling the relative variation of resistance in regard to *R*_0_, second, it is assumed that the effective area for tunneling conduction is held constant regardless of *σ*, hence, the quotient *A*(*σ*)/*A*_0_ is not included because Zhang et al. assumed that *A*(*σ*) = *A*_0_, and third, Equation (17) predicts a voltage-independent behavior for *R*(*σ*).

The second model was obtained on the basis of the Nutting equation that relates sensor strain with time:

(18) $$\varepsilon (t)=\varepsilon_{0}+\psi \sigma t^{n}$$

where *ε*_0_ is the original strain immediately upon the application of stress, *ψ* and *n* are constants estimated on an empirical basis from a known-input stress. The time dependence of *s* under conditions of constant stress can be calculated from:

(19) $$s(t)=s_{0}\left[1-\varepsilon (t)\right]$$

Finally, the second model can be obtained by combining Equations (9) and (19) in regard to *R*_0_:

(20) $$\frac{R(t)}{R_{0}}=\left(\frac{1-\varepsilon (t)}{1-\varepsilon_{0}}\right)\exp\left[-\frac{4\pi }{h}\sqrt{2mV_{a}}s_{0}\left[\varepsilon (t)-\varepsilon_{0}\right]\right]$$

Explicit time dependence on Equation (20) is possible by substituting Equation (18) on the time-dependent strain, *ε*(*t*). Likewise, the expression for *s*_0_ can be also included in the second model from Zhang et al. as next:

(21) $$\frac{R(t)}{R_{0}}=\left(1-\frac{\psi \sigma t^{n}}{1-\varepsilon_{0}}\right)\exp\left\{-\frac{4\pi }{h}\sqrt{2mV_{a}}\left[{\left(\frac{\pi }{6\theta }\right)}^{1/3}-1\right]\psi \sigma Dt^{n}\right\}$$

### 3.2. The model of Wang et al. [23]

Wang et al. have developed several models to describe the piezoresistance behavior of polymer composites [1,6,7,23,38]. However, the contribution from [23] is the most relevant because it comprises multiple parameters in regard to the spatial distribution of the conductive nanoparticles, for such reason, such contribution is here described.

**Figure 7:**
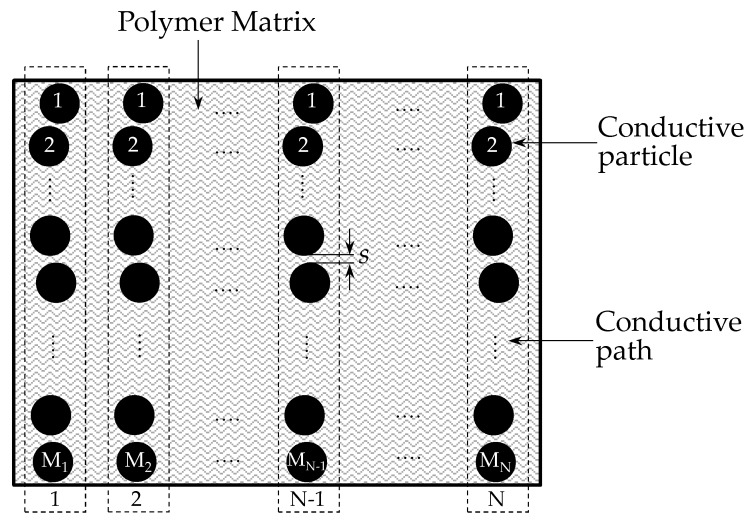
Schematic representing the particle distribution along the insulating polymer matrix. Theoretical description proposed by Wang et al. [23].

Given an insulating polymer matrix with *N* paths for current conduction, the following equation can be stated for the electrical resistance of a polymer composite:

(22) $$R(\sigma )=\frac{1}{N(\sigma )}\left[(K-1)\frac{2sh^{2}}{3e^{2}A\sqrt{2mV_{a}}}\exp\left(4\pi sh^{-1}\sqrt{2mV_{a}}\right)\right]$$

where *s* stands for the inter-particle separation and *K* represents the total number of particles along a conductive path as shown in Figure 7. Equation (22) was obtained from (9) with the addition of the operands *K* and *N* to take into account the multiple conductive paths. Note that Equation (22) embraces dependence upon the area *A*, and also, dependence upon the number of conductive paths, *N*. However, the dependence upon *A* is later discarded by Wang et al. as the final model assumes the relative variation of resistance (*R_r_*(*σ*)) as:

(23) $$R_{r}(\sigma )=\frac{R(\sigma )}{R(0)}=\frac{R_{s}(\sigma )/N(\sigma )}{R_{s}(0)/N(0)}$$

where *R_s_*(*σ*) is the electrical resistance of a single conductive path under stress. On the other hand, the following magnitudes, related to the rest state, *R_s_*(0), *R*(0), *N*(0) are the resistance of a single conductive path, the resistance of the whole polymer composite and the number of conductive paths, respectively. Note that the dependence on *A* has been discarded on Equation (23), but the dependence on the quotient *N*(*σ*)/*N*(0) remains. To authors’ criteria; this is the main contribution proposed by Wang et al. [23] since the quotient *N*(*σ*)/*N*(0) was useful to model the response of polymer composites working on the basis of percolation, readers may refer to Equation (21) at [23]. It must be remarked that the percolation phenomenon is not addressed in this article, because quantum tunneling is the predominant conduction mechanism for the A201-1 and FSR 402 sensors.

Finally, note from Equation (23) that a voltage-independent behavior is assumed, and just as the previous formulation from Zhang et al. [22], the model relies upon experimental measurements of the electrical resistance at sensor rest, *R*(0).

### 3.3. The model of Kalantari et al. [9]

The theoretical formulation of Kalantari et al. is quite similar to the model of Zhang et al*.* [22], with the difference that the contact resistance, *R_Con_*, was included in the model. By combining the expressions for the contact resistance, Equations (10) and (11), with the Zhang et al. formulation at Equation (17), the initial proposal from Kalantari et al. can be obtained as:

(24) $$R_{total}=\frac{\rho_{1}+\rho_{2}}{2}\sqrt{\frac{\pi H}{F}}+R_{0}(1-\varepsilon )\exp\left[-\frac{4\pi }{h}\sqrt{2mV_{a}}D\varepsilon \left[{\left(\frac{\pi }{6\phi }\right)}^{1/3}-1\right]\right]$$

where all the symbols from Equation (24) have the same meaning as from the Zhang et al. formulation in Section 3.1. A description for the symbols *ρ*_1_*, ρ*_2_, *F* and *H* was already presented on Section 2.3. 

**Figure 8:**
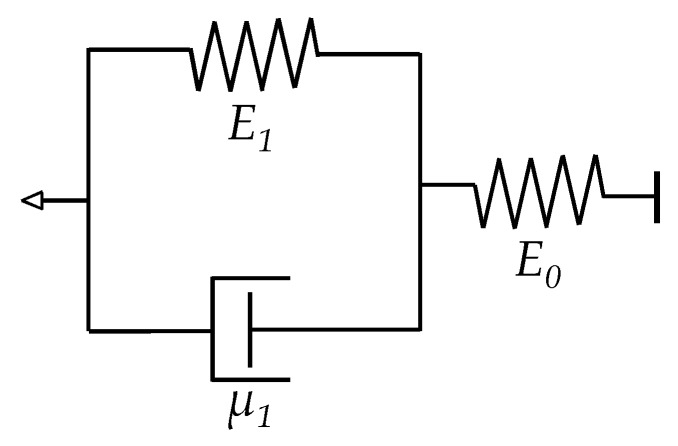
Rheological model for a Zener element.

The main difference between the formulation of Zhang et al. and Kalantari et al. is that the latter employed the Zener rheological model [24] to describe the time dependence of strain, *ε*, whereas the former employed the nutting equation from Equation (18). The relationship between strain, *ε*, and force, *F*, for a Zener element is given by:

(25) $$\varepsilon (t)=\frac{F}{A_{FSR}E_{0}}+\frac{F}{A_{FSR}E_{1}}\left(1-\exp(-E_{1}t/\mu_{1})\right)$$

where *E*_0_, *E*_1_ and *μ*_1_ are the elasticity and viscosity constants from the Zener element shown in Figure 8. The factor *A_FSR_* is the corresponding area of the force sensor, which must not be confused with the effective area for tunneling conduction, *A*. Readers may refer to Appendix A for more details regarding this topic. Finally the model proposed by Kalantari et al. can be obtained by replacing *ε* in Equation (24) for the time-dependent *ε*(*t*) as in Equation (25). The final expression is presented below:

(26) $$\begin{array}{l} R_{total}=\frac{\rho_{1}+\rho_{2}}{2}\sqrt{\frac{\pi H}{F}}+R_{0}\left\{1-\frac{F}{A_{FSR}E_{0}}+\frac{F}{A_{FSR}E_{1}}\left(1-\exp(-E_{1}t/\mu_{1})\right)\right\}\cdot \\ \exp\left\{-\frac{4\pi }{h}\sqrt{2mV_{a}}D\left[{\left(\frac{\pi }{6\phi }\right)}^{1/3}-1\right]\left[\frac{F}{A_{FSR}E_{0}}+\frac{F}{A_{FSR}E_{1}}\left(1-\exp(-E_{1}t/\mu_{1})\right)\right]\right\} \end{array}$$

Following with the trends of Zhang et al. [22] and Wang et al. [23], the model from Kalantari et al. also relies upon measurements of the electrical resistance at sensor rest, see the *R*_0_ factor at Equations (24) and (26). Likewise, the input voltage, *U*, and the area *A* are not considered as parameters in either equation.

## 4. Proposal of a New Model for the Current-Voltage and the Stress-Resistance Relationships of FSRs

With the aim of avoiding any possible confusion, a different nomenclature is henceforth used in the derivation of the proposed model; this was mandatory because new mathematical expressions are introduced in this section for the resistance of the polymer composites, *R_Pol_*, and the contact resistance, *R_Con_*. It must be remarked that multiple expression for *R_Pol_* were presented in Section 3 as a part of the models from Zhang et al. [22], Wang et al. [23] and Kalantari et al. [9]. Likewise, an expression for *R_Con_* was already given in Equation (11).

The formulation from Equation (10) is the start point for the proposal of the new model, but a different nomenclature is henceforth used below:

(27) $$R_{FSR}=2R_{c}+R_{bulk}$$

where *R_FSR_* is the total resistance across the FSR comprising a series connection between the bulk resistance (*R_bulk_*) and the contact resistance (*R_c_*). The same sketch from Figure 5 can be used to represent the series connection of *R_c_* and *R_bulk_* with the proviso that *R_Con_* has been re-labeled as *R_c_* and *R_Pol_* has been re-labeled as *R_bulk_*. Note that the same current *I* flows through both resistances. Hence, the following expression is yielded if Equation (27) is multiplied by *I*:

(28) $$V_{FSR}=2V_{Rc}+V_{bulk}$$

In previous sections *U* has been employed to refer to the input voltage; *U* has been also used for the voltage across the thin insulating film, such a designation is enough for [9,21,22,23], but for the sake of this paper, it turns out to be insufficient because when operating on the basis of Equations (27) and (28), the input voltage—henceforth labeled as *V_FSR_*—is split between the voltage across the polymer composite (*V_bulk_*), and the voltage across the contact resistance (*V_Rc_*). For such reason, *U* is no longer used in this article, but *V_FSR_*, *V_bulk_* and *V_Rc_* are used instead.

### 4.1. New Proposed Model for the Resistance of Polymer Composites, R_bulk_, and the Effective Area, A

The proposed model embraces two additional parameters: the stress-dependent area, *A*, and the voltage across the polymer composite, *V_bulk_*. It must be stated that the distinction presented in Section 2.2 in regard of the difference between polymer composites and FSRs. In this Section, the contact resistance existing between the electrodes and the conductive particles is deliberately omitted, but in Section 4.3, a combined model for *R_c_* and *R_bulk_* is presented. 

Let *A*(*σ*) be the effective area for the electrons to cross the potential barrier as a function of the applied stress with the following general form:

(29) $$A(\sigma )=A_{0}+f(\sigma )$$

where *A*_0_ is the effective area for tunneling conduction at rest state, and *f*(*σ*) is a stress dependent function that describes the increase in the number of conduction paths—and hence of the effective area—as the applied stress increases; such a phenomenon was previously depicted in Figure 6. for the case of FlexiForce and Interlink sensors. The best suited form for *f*(*σ*) was found to be a power function:

(30) $$f(\sigma )=A_{1}\sigma^{A_{2}}$$

It must be pointed out that Equation (30) has been proposed after testing several models on the experimental data from the A201-1 and FSR 402 sensors. Thus, Equation (30) cannot be assumed as a general model for the effective area of any piezoresistive sensor. Different forms of *f*(*σ*) could be proposed for custom-made sensors and for other sensor brands. However, the power-law from Equation (30) is quite similar to the behavior of the contact resistance; this similarity is later discussed in Section 4.2.

Variations in *V_bulk_* modify *R_bulk_* in a highly non-linear fashion as previously shown in the simulation plot of Figure 4b. When *V_bulk_* is in the millivolt range, *R_bulk_* exhibits an ohmic behavior. When *V_bulk_* is increased beyond the millivolt threshold, *V*th, a decrease in *R_bulk_* is expected for incremental values of *V_bulk_*. Finally, when *V_bulk_* is greater than the height of the potential barrier, *V_a_/e*, a small increment in *V_bulk_* yields a dramatic increase in sensor current. These statements have been experimentally demonstrated by Fisher and Giaever [20]. 

In a typical force sensing application, *V_FSR_* is usually within the range (300 mV–5 V) [13,28]. It is later demonstrated in Section 5 that under such circumstances, the value of *V_bulk_* is typically larger than *V_a_/e*, and thus, Equation (8) can be taken as the equation for describing the current conduction in the polymer composite. Note that a close form for *R_bulk_* is not possible in such interval and only an *I-V_FSR_* relationship can be formulated. When *V_FSR_* is within the range (0 V–150 mV), *R_bulk_* has an ohmic behavior because *V_bulk_* is typically lower than *V*th. Thus, a close form for *R_bulk_* is possible in this interval. Finally, when *V_FSR_* is within the range (150 mV–300 mV) *V_bulk_* is greater than *V*th but smaller than *V_a_/e*, under such circumstances, Equation (7) describes the *I-V_FSR_* relationship of the polymer composite.

Unfortunately, the previously mentioned intervals are approximated because *V_bulk_* is a function of *V_FSR_* and *R_c_*, see Equation (28). Hence, absolute intervals cannot be defined for *V_bulk_* because *R_c_* varies from one sensor to another, i.e., there are not two identical sensors. For such reason, the piecewise model for the polymer composite must be stated in terms of: *V_bulk_* = *V_FSR_* − 2·*I*·*R_c_*.

If *V_FSR_* − 2·*I*·*R_c_* < *V*th Equations (6), (16), (29), (30), (A2) and (A3) are combined as next:

(31) $$R_{bulk}=\frac{2s_{0}(1-\sigma /M)}{3\left[A_{0}+A_{1}\sigma^{A_{2}}\right]\sqrt{2mV_{a}}}{\left(\frac{h}{e}\right)}^{2}\exp\left(\frac{4\pi }{h}s_{0}(1-\sigma /M)\sqrt{2mV_{a}}\right)$$

where Equation (31) is valid only when *V_FSR_* is approximately within the range (0 V–150 mV).

If *V*th *< V_FSR_* − 2·*I*·*R_c_* < *V_a_/e* Equations (7), (16), (29), (30) and (A2) are combined as next:

(32) $$\begin{array}{l} I=\frac{\left[A_{0}+A_{1}\sigma^{A_{2}}\right]e}{2\pi hs_{0}^{2}{\left(1-\sigma /M\right)}^{2}}\{\left(V_{a}-\frac{eV_{bulk}}{2}\right)\exp\left[-\frac{4\pi }{h}s_{0}(1-\sigma /M)\sqrt{2m\left(V_{a}-\frac{eV_{bulk}}{2}\right)}\right]\\ \;\;\;\;\;\;\;\;-\left(V_{a}+\frac{eV_{bulk}}{2}\right)\exp\left[-\frac{4\pi }{h}s_{0}(1-\sigma /M)\sqrt{2m\left(V_{a}+\frac{eV_{bulk}}{2}\right)}\right]\} \end{array}$$

where Equation (32) is valid only when *V_FSR_* is approximately within the range (150 mV–300 mV)

Finally, if *V_FSR_* − 2·*I*·*R_c_* > *V_a_/e* Equations (8), (16), (29), (30) and (A2) are combined as next:

(33) $$\begin{array}{l} I=\frac{2.2e^{3}V_{bulk}^{2}\left[A_{0}+A_{1}\sigma^{A_{2}}\right]}{8\pi hV_{a}s_{0}^{2}{\left(1-\sigma /M\right)}^{2}}\{\exp\left[-\frac{8\pi s_{0}(1-\sigma /M)}{2.96heV_{bulk}^{2}}\sqrt{2mV_{a}^{3}}\right]\\ \;\;\;\;\;\;\;\;-\left(1+\frac{2eV_{bulk}}{V_{a}}\right)\exp\left[-\frac{8\pi s_{0}(1-\sigma /M)}{2.96heV_{bulk}}\sqrt{2mV_{a}^{3}\left(1+\frac{2eV_{bulk}}{V_{a}}\right)}\right]\} \end{array}$$

where Equation (33) is valid only when *V_FSR_* is greater than 300 mV.

Note that Equations (31)–(33) notably vary from the models previously described on Section 3. The Equations (31)–(33) impose a voltage-dependent and area-dependent behavior for the current flowing through the polymer composite. Likewise, they do not rely upon experimental measurements of the electrical resistance at rest state, *R*_0_.

### 4.2. New Proposed Model for the Contact Resistance (R_c_) 

In Section 2.3, a model for the contact resistance was presented based upon previous results from Kalantari et al. [9]. Equation (11) predicts a reduction in the contact resistance in an inverse square root fashion of the applied force. However, as later demonstrated in Section 5, the application of such a model is not suitable, at least for the FlexiForce and Interlink sensors. For this reason, the following model is proposed:

(34) $$R_{c}=R_{par}+\frac{R_{c}^{0}}{\sigma^{k}}$$

Previous Equation can be stated in terms of the applied force, *F*, to allow comparison with the formulation from Kalantari et al.:

(35) $$R_{c}=R_{par}+\frac{R_{c}^{0}}{{\left(F/A_{FSR}\right)}^{k}}$$

where *A_FSR_* is the physical area of the FSR, and $R_{c}^{0}$ is the value of the contact resistance at rest state.

Note from Equations (34) and (35) that an infinite resistance is expected when *σ = F = 0*. In practice, it does not occur because the sensor is preloaded with a small force imposed by the sensor encapsulating material which is tightly bonded around the sensor edges. An offset can be added to Equations (34) and (35) in the form of *σ* + *σ*_0_ or *F* + *F*_0_, but typically, the values of *σ*_0_ and *F*_0_ are negligible when compared with the exerted forces, so, they have not been included in the aforementioned equations.

The resistance of the conductive particles, *R_par_*, is included in Equations (34) and (35) because at the nano- and microscopic levels, the conductive particles behave as quantum point contacts, and thus, the classical definition of resistivity does not hold. This occurs when the length of the particle is comparable with 2π/*K_f_*, where *K_f_* is the Fermi wavefactor. Under such circumstances, the conductance of a nanoparticle is quantized in terms of the conductance quantum *G*_0_:

(36) $$G_{0}=\frac{2e^{2}}{h}=7.74\cdot 10^{-5}S$$

The conductance increments occur in integer multiples of the particle length (*L_par_*) as next:

(37) $$L_{par}=n\frac{2\pi }{K_{f}}$$

where *n* = 0, 1, 2… and *K_f_* is given by:

(38) $$K_{f}=\frac{\sqrt{2mE_{f}}}{\hbar }$$

with *E_f_* standing for the Fermi energy of the particle. The phenomenon of quantum point contacts was discovered by independent studies carried out by Van Wees et al. [39] and Wharam et al. [40], but a comprehensive explanation of the phenomenon is addressed by Timp [41]. In brief, for discrete increments of *L_par_*, the particle’s conductance grows in a discrete fashion of *n*·*G*_0_. Only for sufficiently large values of *n*, the conductance follows a continuous-classical-variation.

To the best of authors’ knowledge, the proposal of quantum point contacts for the conductive nanoparticles has not been considered before by any author in the polymer composite field [33,34,42,43,44]. This is an important contribution since it complements previous models from Zhang et al. [22], Wang et al. [23] and Kalantari et al. [9] in which they assumed that *R_par_* is negligible when compared to *R_pol_*, see Equation (13). Moreover, the experimental results from Section 5 support the hypothesis that *R_par_* cannot be neglected.

On the other hand, the form $R_{c}^{0}/\sigma^{k}$ from Equation (34) and $R_{c}^{0}/{\left(F/A_{FSR}\right)}^{k}$ from Equation (35) have been previously reported for the constriction (contact) resistance of materials with different sizes. In general, the constriction resistance follows power-laws as stated by Shi et al. [45] and Mikrajuddin et al. [36]. So, the form of Equations (34) and (35) is plausible as it models the series connection between the contact resistance—following power-laws—and the nanoparticle resistance. As previously stated, the underlying basis for the contact resistance can be found on the plastic and elastic interactions occurring between the conductive particles and the sensor electrodes at a microscopic level [35]. Similarly, the increment in the effective area, *A*, follows a power-law function as previously presented on Equations (29) and (30); this is not a coincidence as the behavior of *R_c_* and *A* are tightly related, see Section 2.3 and Figure 6.

In Section 5.2.1, experimental data are fitted to Equation (34) with good results. However, the model for the contact resistance can be further generalized, because as previously stated in Equation (12) the resistance of the conductive particles is computed from *L*·*R_par_*/*S*, where *L* and *S* are the number of series-connected and parallel-connected particles, respectively. Nonetheless, when the applied stress increases more current paths are formed, and therefore, the number of parallel-connected particles, *S*, also grows. This implies that *S* should have the form *S*(*σ*), but for simplification purposes and considering the good results obtained, the proposed model from Equation (34) is henceforth used when modelling the contact resistance.

Finally, note that Equations (34) and (35) predict a voltage independent behavior for the contact resistance. This statement is experimentally demonstrated on Section 5 on the basis of applying large values of *V_FSR_* at different stresses.

### 4.3. Final Proposed Model for the I-V_FSR_ Relationship of Force Sensing Resistors, FSRs

The final proposed model for the *I-V_FSR_* relationship of FSRs is based on combining the expression for the *I-V_FSR_* relationship of polymer composites, Equations (31)–(33), with the expression for the contact resistance.

For the sake of providing a consistent formulation with previous sections on this article, the final model is stated in terms of stress; hence Equation (34) is chosen as the mathematical expression for the contact resistance. The framework for combining both expressions is based upon Equations (27) and (28).

Considering that multiple symbols are embraced in the proposed model, Table 1 summarizes the symbols employed by the authors while providing a comparison with previous notation from Zhang et al. [22], Wang et al. [23] and Kalantari et al. [9]. The proposed model is presented next using piecewise functions in regard of *V_bulk_*, where *V_bulk_* = *V_FSR_* − 2·*I*·*R_c_* according to Equation (28).

If *V_FSR_* − 2·*I*·*R_c_* < *V*th, Equations (27), (31) and (34) are combined to obtain:

(39) $$R_{FSR}=2R_{par}+\frac{2R_{c}^{0}}{\sigma^{k}}+\frac{2s_{0}(1-\sigma /M)}{3\left[A_{0}+A_{1}\sigma^{A_{2}}\right]\sqrt{2mV_{a}}}{\left(\frac{h}{e}\right)}^{2}\exp\left(\frac{4\pi }{h}s_{0}(1-\sigma /M)\sqrt{2mV_{a}}\right)$$

If *V*th *< V_FSR_* − 2·*I*·*R_c_* < *V_a_/e*, Equations (28) and (32) are merged, so that *V_bulk_* is stated in terms of *V_FSR_* and *R_c_* as next:

(40) $$\begin{array}{l} I=\frac{\left(A_{0}+A_{1}\sigma^{A_{2}}\right)e}{2\pi hs_{0}^{2}{\left(1-\sigma /M\right)}^{2}}\{\left(V_{a}-\frac{e(V_{FSR}-2R_{c}I)}{2}\right)\exp\left[-\frac{4\pi }{h}s_{0}(1-\sigma /M)\sqrt{2m\left(V_{a}-\frac{e(V_{FSR}-2R_{c}I)}{2}\right)}\right]\\ \;\;\;\;\;\;\;\;\;-\left(V_{a}+\frac{e(V_{FSR}-2R_{c}I)}{2}\right)\exp\left[-\frac{4\pi }{h}s_{0}(1-\sigma /M)\sqrt{2m\left(V_{a}+\frac{e(V_{FSR}-2R_{c}I)}{2}\right)}\right]\} \end{array}$$

If *V_FSR_* − 2·*I*·*R_c_* > *V_a_/e*, Equations (28) and (33) are merged, so that *V_bulk_* is stated in terms of *V_FSR_* and *R_c_* as next:

(41) $$\begin{array}{l} I=\frac{2.2e^{3}{\left(V_{FSR}-2R_{c}I\right)}^{2}\left[A_{0}+A_{1}\sigma^{A_{2}}\right]}{8\pi hV_{a}s_{0}^{2}{\left(1-\sigma /M\right)}^{2}}\{\exp\left[-\frac{8\pi s_{0}(1-\sigma /M)}{2.96he{\left(V_{FSR}-2R_{c}I\right)}^{2}}\sqrt{2mV_{a}^{3}}\right]\\ \;\;\;\;\;\;\;\;\;\;-\left(1+\frac{2e(V_{FSR}-2R_{c}I)}{V_{a}}\right)\exp\left[-\frac{8\pi s_{0}(1-\sigma /M)}{2.96he(V_{FSR}-2R_{c}I)}\sqrt{2mV_{a}^{3}\left(1+\frac{2e(V_{FSR}-2R_{c}I)}{V_{a}}\right)}\right]\} \end{array}$$

where *R_c_* is given by Equation (34). 

Equations (39)–(41) are stated in terms of the universal constants: *m*, *e*, *h*, specific properties from the polymer and the nanoparticles: *s*_0_, *M*, *V_a_*, *A*_0_, *A*_1_, *A*_2_, and in terms of the contact resistance, *R_c_*. This last term can be independently estimated by re-arranging Equation (28):

(42) $$R_{c}=\frac{V_{FSR}-V_{bulk}}{2I}$$

For very large values of *V_FSR_*, the *V_bulk_* term from Equation (42) can be set to 0 as an initial approximation because *I* grows with the square of *V_bulk_*—see Equations (33) and (41)—whereas the voltage drop across the contact resistance, *V_Rc_*, grows linearly with the sensor current. This implies in practice, that for a constant stress, an increment in *V_FSR_*—and hence on sensor current—yields a small increment of *V_bulk_*, whereas most of the additional *V_FSR_* actually drops in the contact resistance, i.e., for large values of *V_FSR_*, *R_c_* dominates because *R_bulk_* is diminished, see Figure 4b. Finally, Equation (42) can be approximated to the following expression for sufficiently large values of *V_FSR_*:

(43) $$R_{c}\approx \frac{V_{FSR}}{2I}$$

Equation (43) is very useful in practice because it allows an approximated estimation of the contact resistance in an empirical basis. Moreover, Equation (43) does not require information from the materials employed during the manufacturing process of the polymer composites. An iterative process that yields accurate results for *R_c_* is addressed in the next Section.

## 5. Experimental Setup, Results and Discussion

In this section, the experimental setup for data gathering is described, followed by the presentation of the experimental results and discussion.

### 5.1. Experimental Setup

**Figure 9:**
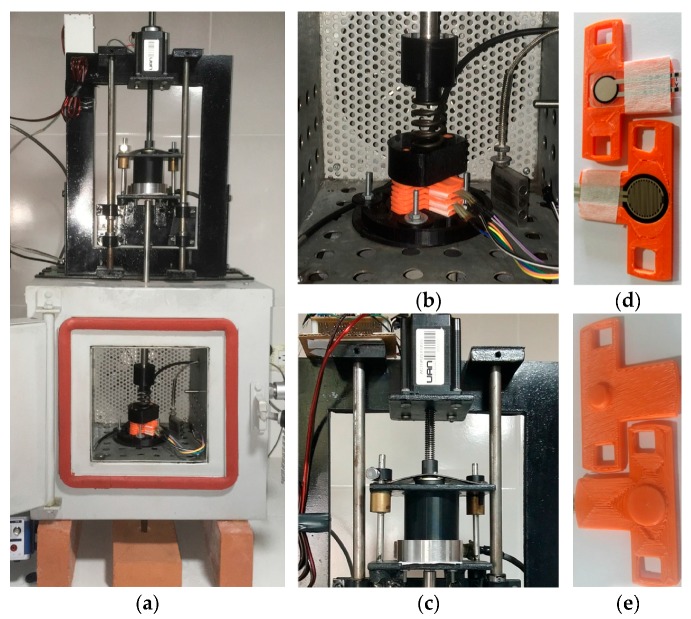
Mechanical setup for gathering sensor data. (**a**) Overview of the testbench; (**b**) Zoom-in picture depicting the sensors location inside the chamber and the spring for mechanical compliance; (**c**) Zoom-in picture showing the linear stepper motor and the LCHD-5 load cell, manufactured by Omega Engineering (Norwalk, CT, USA); (**d**) Top and (**e**) bottom views of the custom-made sensor holders and pucks, respectively; Sensors are harnessed using paper tape as in (**d**).

The experimental setup for sensor testing is summarized on Figure 9. It comprised a custom-made temperature chamber with force ventilation, and a linear stepper motor to exert dynamic forces over the FSRs, see Figure 9a. Mechanical compliance was made possible by adding a spring between the motor and the sensors as shown in Figure 9b. The force loop was closed using a LCHD-5 load cell, manufactured by Omega Engineering (Norwalk, CT, USA). The LCHD-5 load cell has a nominal capacity of 22 N, see Figure 9c. Nonetheless, some tests required a larger capacity load cell, so the LCHD-25 with a nominal range of 111 N was employed for the tests of Section 5.4.

In order to avoid sensor displacement, and to ensure an evenly force distribution along the Sensor Sensitive Area, SSA, custom-made pucks were built as shown in Figure 9d,e. The round pucks had diameters of 7.3 mm. and 15.3 mm. for the A201-1 and the FSR 402, respectively. Hence, the values of *A_FSR_* were equal to: 41.85 mm^2^ and 183.85 mm^2^ for each sensor, respectively.

The temperature along all the tests reported in this article was set to 25 °C ± 1 °C, the absolute error of 1 °C of is the sum of temperature gradient along the chamber plus PT100 sensor error. Temperature control and monitoring are important because later work from Simmons demonstrated that temperature influences the current conduction through thin insulating layers [46].

**Figure 10:**
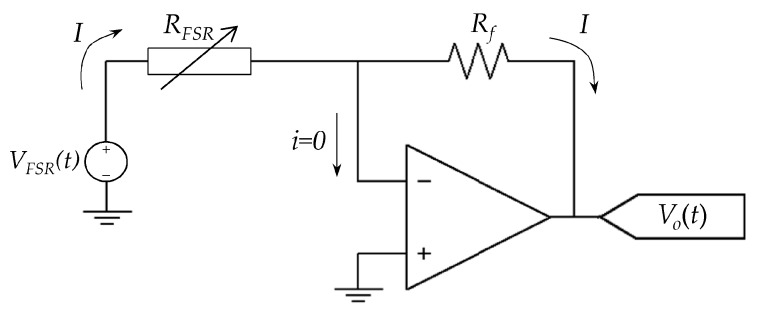
Driving circuit for the FSRs.

The electrical setup for sensor driving and data collection is summarized on Figure 10. The driving circuit is based on an amplifier in inverting configuration with the following general formula:

(44) $$R_{FSR}=-\frac{V_{FSR}(t)}{V_{o}(t)}R_{f}$$

where *R_f_* is the feedback resistor, *V_o_* is the amplifier output voltage and *R_FSR_* is the resistance of the FSR as previously defined in Equation (27). Note that the input voltage *V_FSR_*(*t*) has been stated in Equation (44) as a time-dependent function; this was done so because in some tests *V_FSR_*(*t*) was set as a sawtooth function, whereas in others, *V_FSR_*(*t*) was set as a DC voltage.

An important consideration must be stated from the circuit of Figure 10; it ensures that the voltage across the sensor is effectively controlled at all times, i.e., *V_FSR_*(*t*) is fixed and independent of the applied stress, but only sensor current is modified by *σ*. Likewise, the sensor current can be estimated from:

(45) $$I=-\frac{V_{o}(t)}{R_{f}}$$

The setup from Figure 10 notably differs from previous experimental setups of Kalantari et al. [9], Zhang et al. [22] and Wang et al. [6,23] because they employed voltage dividers or multimeters to readout *R_FSR_*. Under such experimental setups, *V_FSR_* is modified as the applied force changes. An explanation on how a digital multimeter operates can be found in [47]; in brief, a digital multimeter injects a fixed (known) current to the unknown resistance, and then, the voltage drop across the resistance is measured on the basis of the Ohm’s law. 

Finally, data acquisition was made from the board NI9205 installed in a CRIO-9035 system; the NI9205 has a 16 bit ADC with customizable nominal ranges starting at ±200 mV up to ±10 V. The *V_FSR_* range starts from a few millivolts up to 26 V; such a wide range was required for *V_FSR_* to allow an estimation of *R_c_* on the basis of Equation (43). Moreover, in some tests *V_FSR_* was set as high as 58 V, see Section 5.4.

### 5.2. Experimental Results

**Figure 11:**
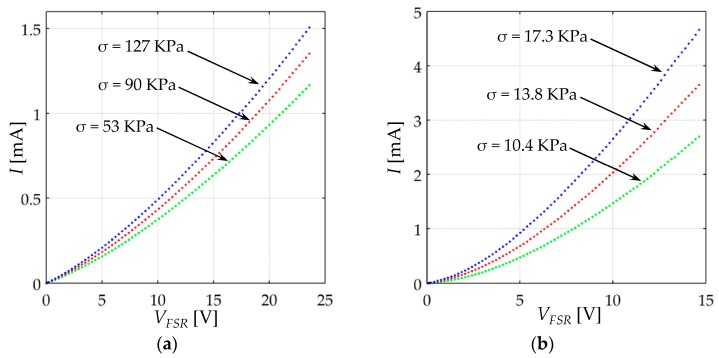
Current-Voltage (*I-V_FSR_*) relationship at different stresses for the sensors: (**a**) FlexiForce A201-1 and (**b**) Interlink FSR 402.

Figure 11 shows the *I-V_FSR_* relationship for the FlexiForce A201-1 and Interlink FSR 402 sensors. Note that the curves for every stress are clearly non-linear; this behavior has been previously reported by an authors’ previous study [48], but up to now, the underlying basis of such non-linearity have not been explained.

**Figure 12:**
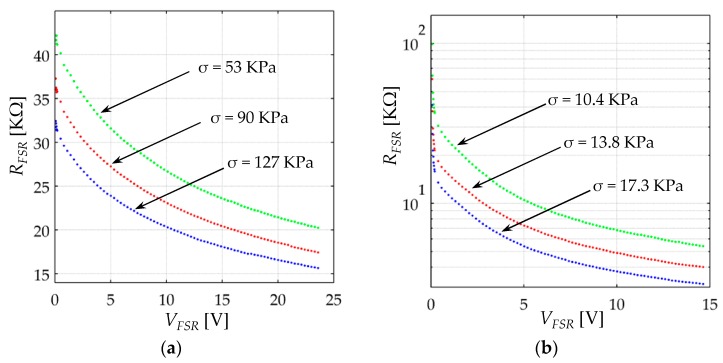
Electrical Resistance (*R_FSR_*) at different stresses for the sensors: (**a**) FlexiForce A201-1 and (**b**) Interlink FSR 402.

Sensor resistance, *R_FSR_*, was computed for every applied stress on the basis of Equation (44), and the resulting data are plotted on Figure 12 for both sensors. Note that the resistance is influenced by *V_FSR_* following a similar trend to that predicted by Simmons [21], and Fisher and Giaever [20]. The responses from Figure 12 cannot be predicted by either Zhang et al. [22], Wang et al. [23] or Kalantari et al. [9], because they all assumed *R_FSR_* as a voltage-independent magnitude, see Equations (17), (22) and (24). 

It must be highlighted from Figure 12 that *R_FSR_* is influenced by the voltage across the sensor, *V_FSR_*. Hence, if *V_FSR_* is not held constant, different *R_FSR_* values can be obtained for the same device under the same loading conditions. For this reason, measuring *R_FSR_* using a multimeter, a Wheatstone bridge or a voltage divider is not recommended. Conversely, the experimental setup from Figure 10 ensures that the voltage across the sensor is constant and independent of the applied stress. In fact, memristive devices benefit from the non-linear voltage-dependent behavior of the tunneling resistance for exhibiting memory curves [49].

Note from the plots of Figure 12, that as *V_FSR_* increases, *R_FSR_* asymptotically approaches to a low-limit value given by the contact resistance, *R_c_*. The following iterative method is proposed and tested to estimate *s*_0_, *M*, *V*th, *V_a_*, *A*_0_, *A*_1_, *A*_2_, and *R_c_* for a given sensor:

(i) Set *V_FSR_*(*t*) as the largest possible DC voltage that avoids sensor heating. Apply different stresses to the sensors and estimate sensor current, *I*, for every applied stress using Equation (45). In practice, it was set *V_FSR_*(*t*) = 26 V, *σ* = 300 KPa for the A201-1 sensor and *V_FSR_*(*t*) = 15 V, *σ* = 34 KPa for the FSR 402 sensor.
(ii) Apply a sawtooth signal in *V_FSR_* starting from tens of millivolts up to a sufficiently large voltage that ensures *V_bulk_* > *V_a_/e*. In practice, *V_FSR_ = 0*:*∆V*:*V_max_* with *∆V* = 20 mV and *V_max_* equal to 10 V for the A201-1 and 8 V and the FSR 402. Repeat the process at different stresses and estimate sensor current for each pair of *V_FSR_*, *σ*. Determine the millivolt threshold, *V*th, from the experimental data. *V*th defines a lower bound for *V_a_* in the surface fit which will be explained later. A smaller voltage increment, *∆V*, may be necessary to accurately determine *V*th.
(iii) Using the data from (i), calculate *R_c_* using Equation (43) in the first iteration. For iterations two and on, solve Equation (41) to find *V_bulk_*, and then estimate *R_c_* from Equation (42).
(iv) By using fitting tool software, perform a surface fit for the experimental data from (ii). Use the piecewise functions from Equations (39)–(41) as the theoretical model, and *s*_0_, *M*, *V_a_*, *A*_0_, *A*_1_, *A*_2_ as the unknown parameters. Software tools such as Matlab^®^ and Python allow surface fitting of piecewise functions with unknown limits; this is necessary as *V_a_* is both: a parameter of Equations (40) and (41), and also, the limit that defines the transition between both equations. Set the experimentally measured *V*th from (ii) as the limit between Equations (39) and (40), and set *V_a_/e* > *V*th as the lower bound for *V_a_*. Supplementary material included in this article demonstrates how this fit was performed by using Matlab R2014a.
  Recalling previous studies, *s*_0_ is usually in the nanometer range [20,21], and *M* is in the range of tens to hundreds of Mega Pascals for rubber and elastomers. FlexiForce and Interlink sensors are manufactured from such materials [14,15,17,18].
(v) Repeat steps (iii)–(v) until convergence on the parameters *s*_0_, *M*, *V_a_*, *A*_0_, *A*_1_, *A*_2_ is reached. Similarly, the parameters for *R_c_* are also modified on each iteration.

Once *s*_0_, *M*, *V*th, *V_a_*, *A*_0_, *A*_1_, *A*_2_, and *R_c_* have been estimated, Equations (39)–(41) can be employed to predict sensor current in an FSR given *σ* and *V_FSR_*. The ability to make predictions on the basis of Equations (39)–(41) is one of the most important contributions from this article. The results from the experimental data fitting are next described:

#### 5.2.1. Measurements of the Contact Resistance, *R_c_*

**Figure 13:**
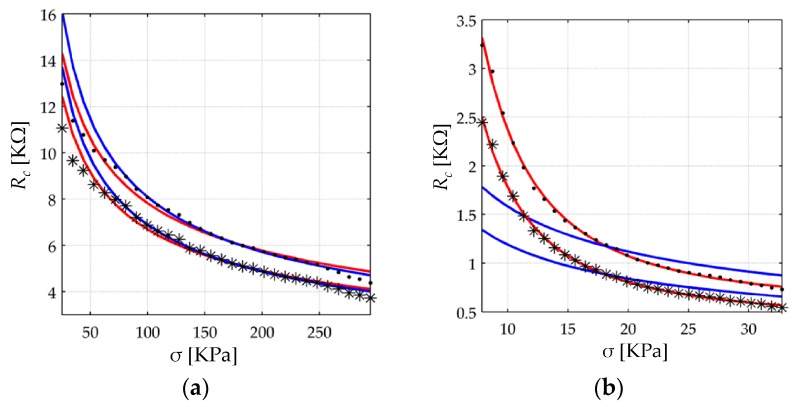
Contact Resistance (*R_c_*) as a function of the applied stress for the sensors: (**a**) FlexiForce A201-1 and (**b**) Interlink FSR 402. Resistance values estimated from step iii) at the first iteration (points) and at the third iteration (asterisk). Trend line description: Kalantari et al. [9] model (blue) and proposed model (red) based on Equation (34).

The contact resistance from step (iii) is plotted on Figure 13 for both sensors. Table 2 presents statistical data and numerical values for the parameters that model *R_c_* on the basis of Equation (34). Note from Figure 13 that the values from the first iteration overestimate *R_c_* as *V_bulk_* was initially assumed as zero in Equation (43). After three iterations, the estimated *R_c_* is somewhat lower. The Kalantari et al. model [9] for the contact resistance has been fitted to the experimental data with the substitution *F* = *σ*·*A_FSR_* in Equation (11). Similarly, the proposed model from Equation (34) has been also fitted to the experimental data. From Figure 13, it is clear that the model of Equation (11) never performed a suitable fit, whereas Equation (34) always did; this is so because of the *R_par_* and *k* parameters which are exclusive of the proposed model. On this topic two statements can be yielded:

First, different power laws have been identified for each sensor; see the *k* parameter in Table 2. Mikrajuddin et al. [36] found that the value of *k* depends upon two factors: the kind of interaction occurring between the probe (nanoparticles) and the plate (electrodes); the second factor is the range of the applied stress. For elastic interactions, *k* switches between discrete values of 1/3 or 2/3; conversely for plastic interactions, *k* switches between discrete values of 1/2 or 1. The *k* values from Table 2 do not match with either value; this suggests that additional phenomena are occurring at a microscopic level; this is later discussed on Section 5.2.3.

Second, the fitting result for the FSR 402 sensor supports the hypothesis of the quantum point contacts in the conductive particles; this has been proposed on Section 4.2. It must be stated that the theory behind quantum point contacts has been thoroughly demonstrated, and thus, it can be taken as a fact [39,40,41]. A Scanning Electron Microscopy (SEM) test could help to confirm that the conductive particles are sufficiently small such to exhibit quantization of conductance. Unfortunately, a SEM analysis was not available at the time of this study. However, by relying upon patent information from the Interlink [17] sensor, the particle size in the FSR 402 is between 0.5 μm and 10 μm; such particle dimensions are sufficiently small to exhibit quantization of conductance. For instance, given silver material for the conductive particles with Fermi level, *Ef*, equal to 5.49 eV, the Fermi wavefactor, *K_f_*, and *L_par_* can be calculated by taking *n* = 1 in Equations (38) and (37), respectively. The conductance increments occur in integer multiples of *L_par_*, and thus, *n* is equal to 958 for a particle of 0.5 μm. Finally, the particle’s conductance can be found from the product *n*·*G*_0_, where *G*_0_ is given by Equation (36) yielding *R_par_* = 13.4 Ω. This resistance may seem a small value, but the net resistance contribution from the particles is computed from the expression *L*·*R_par_*/*S* as in Equation (12).

Unfortunately, the patent currently holding for the FlexiForce sensor [15] does not provide information regarding particle size. Experimental results from Table 2 suggest two possibilities for the A201-1 sensor: either particle sizes are bigger in FlexiForce sensors yielding a larger *n*, and therefore, the product *n*·*G*_0_ is large enough to produce *R_par_ ≈* 0 or the quotient *L*/*S* from Equation (12) is sufficiently small such that *L*/*S ≈* 0.

#### 5.2.2. Measurements of the Millivolt Threshold (*V*th)

As previously addressed on Section 2.2, *V*th is the threshold voltage at which the polymer composite no longer exhibits an ohmic (linear) response. When *V_bulk_* = *V*th, a transition occurs between the piecewise Equations (39) and (40).

In order to determine *V*th, step ii) must be performed at constant stress, *σ*, and the difference between consecutive samples of sensor current, *I*, is calculated for the incremental values of *V_FSR_*. In other words, given *V_FSR_ = 0*: *∆V*: *V_max_*, a resulting *I*[*n*] is obtained at constant *σ* from Equation (45), and thus, *∆I*[*n*] can be computed from:

(46) ΔI[n]=I[n]−I[n−1]

**Figure 14:**
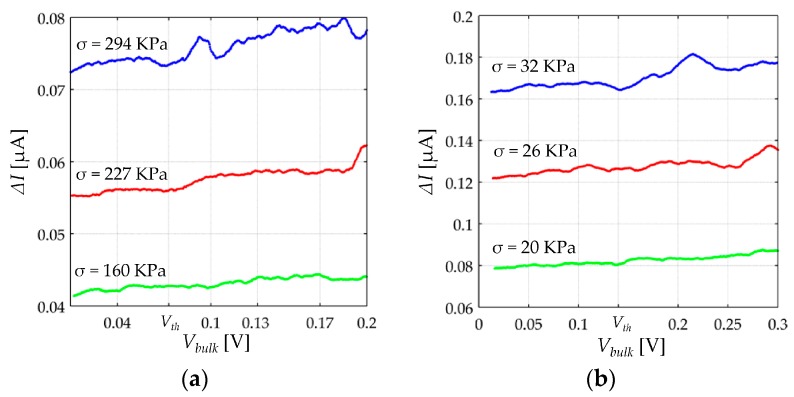
Plots for *∆I*[*n*] as a function of *V_bulk_*[*n*] at different stresses for the sensors: (**a**) FlexiForce A201-1 and (**b**) Interlink FSR 402. *V*th is determined by detecting the slope change of *∆I*[*n*].

When *V_FSR_*[*n*] is within the ohmic region of the FSR, a constant *∆I*[*n*] is observed. *V*th is experimentally determined by finding the lowest value of *V_FSR_* that produces a slope change in *∆I*[*n*]. Unfortunately, Equation (46) is not straightforward in practice because the *∆I* data are too noisy at the millivolt level. Instead, a Savitzky–Golay derivative filter is used and *∆V* was set to 1 mV to obtain sufficient data points. The experimental results are plotted on Figure 14 for both sensors.

It must be remarked that an increment of *V_FSR_* yields an increment in *V_bulk_*, see Equation (28). However, such an increment of *V_FSR_* is split between *V_Rc_* and *V_bulk_*; this implies that *R_c_* must be known before determining *V*th. Fortunately, when operating at the millivolt level, *R_bulk_* is many times larger than *R_c_*, so the contact resistance can be found from Equation (43) with negligible error on the estimated *V*th. The x-axis from Figure 14 matches for *V_bulk_* which is computed from: *V_bulk_*[*n*] = *V_FSR_*[*n*] − 2·*R_c_*·*I*[*n*]. The estimated *V*th are 73 mV and 140 mV for the A201-1 and the FSR 402, respectively. Experimental results support the hypothesis that *V*th is a force-independent quantity just as *V_a_* is; this is a logical result because when subjected to stress, the width of the potential barrier, *s*, is modified, but its height, *V_a_*, is defined from the material properties.

#### 5.2.3. Final Model Resulting from the Data Fit

The main contribution from this article is the proposal and validation of a model for the current conduction of FSRs operating on the basis of quantum tunneling. Given specific conditions of operating voltage, *V_FSR_*, and stress, σ, the proposed model is capable of estimating sensor current, *I*. This is possible from the piecewise Equations (39)–(41).

**Figure 15:**
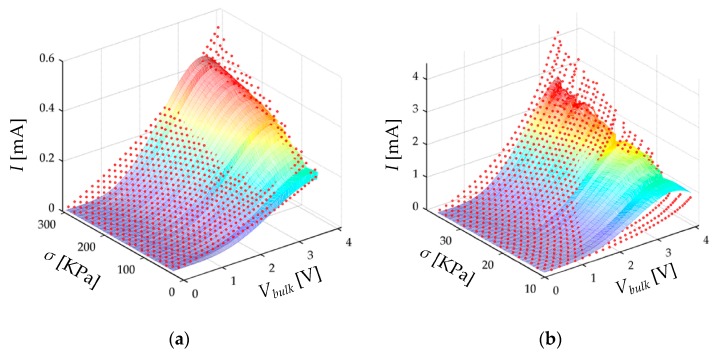
Surface fit and experimental data for the piecewise Equations (39)–(41). Plots for the sensors: (**a**) FlexiForce A201-1 and (**b**) Interlink FSR 402.

As previously stated at Section 5.2, an iterative process must be followed to optimally determine the set of parameters that describe the piezoresistive response of the FSR. The result is a surface fit performed from the experimental data collected on the step ii. The surface fit is shown in Figure 15 with statistical data and parameter information listed on Table 3.

The data fitting processes yielded coefficients of determination, *r*^2^, equal to 0.88 and 0.95 for the A201-1 and the FSR 402 sensors, respectively. Larger values of *r*^2^ could be obtained from the fit, but it must be taken into account that the theoretical model itself is approximated. On this topic, it must be remarked that Simmons [21] made two consecutive approximations during the derivation of his model: first, he solved the Schrödinger equation using the WKB approximation, and second, he assumed as constant some voltage-dependent parameters; this is the case of the *β* factor. Further details on this topic are addressed in Appendix B.

Another reason for the relative low value of *r*^2^ is based on the non-linear least-squares method which was employed for the surface fit. The non-linear least-squares algorithm penalizes large errors, and thus, the parameters resulting from the fit try to minimize the discontinuous output from the fitted model. Recalling Simmons’ statements at [21], the Equations (6)–(8) exhibit more error at the transition voltages occurring at *V_bulk_* = *V*th and at *V_bulk_* = *V_a_/e*. Nonetheless, as *V_bulk_* moves away from *V*th or *V_a_*/*e* the error is quickly diminished. In practice, this implies that the variance of the error frequency distribution is not constant for the Simmons’ model, but the non-linear least-squares algorithm attempts to equalize error variance along the entire data set. The net effect is that there is always an offset between the experimental data and the fitted model, see Figure 15. Similarly, note that no discontinuity is observed, whereas in the simulation plot of Figure 4a the discontinuities are easily observable. However, the accuracy of the proposed model is later tested on Section 5.3 with overall good results.

**Figure 16:**
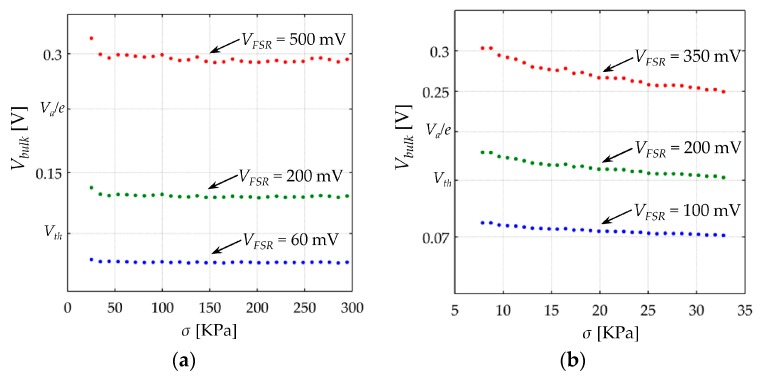
Plot of *V_bulk_* as function of *σ* for different values of *V_FSR_*. The values of *V*th and *V_a_* are listed on Table 3. Data for: (**a**) FlexiForce A201-1 and (**b**) Interlink FSR 402.

The proposed model is plotted on Figure 15 as a function of *σ* and *V_bulk_*. Nonetheless, *V_bulk_* is not straightforward measurable in practice, because *V_bulk_* is a function of *R_c_* and *V_FSR_*; this was previously discussed on Section 4.1. Fortunately, *V_bulk_* can be estimated from *V_FSR_* by following the next steps:

Given the general model for *R_c_* on Equation (34), *V_bulk_* can be estimated for each pair of *σ* and *V_FSR_* by using the parameters from Table 2 and Equation (28), where *V_bulk_* = *V_FSR_* − 2·*I*·*R_c_*. Sensor current, *I*, can be experimentally estimated from Equation (45). Figure 16 shows *V_bulk_* as a function of *σ* for different values of *V_FSR_*. The approximated intervals for the Equations (31)–(33) were obtained following this procedure. Similarly, absolute intervals can be defined for the Equations (39)–(41), but recalling the fact that *R_c_* varies from one sensor to another, the model from Equations (39)–(41) was stated in piecewise intervals using Equation (28). 

Finally, the following analogy can be established between Table 2 and Table 3: for incremental values of stress, the pace for the formation of current paths is faster in the FSR 402 sensor than in the A201-1; this is stemmed from the parameter *A*_2_ in Table 3. It must be remarked that *A*_2_ is part of the model for the effective area *A*, see Equations (29) and (30). Similarly, the contact resistance is faster reduced in the FSR 402 as predicted by the parameter *k* in Table 2; this is not a coincidence because a reduction in the contact resistance is produced by a combination of both phenomena: the pressure dependence on *R_c_* as stated by Mikrajuddin et al. [36], and the formation of new current paths as predicted by Equations (29) and (30). These two phenomena occur simultaneously and explain why none of the Mikrajuddin power laws (1/3, 2/3, 1/2 and 1) are experimentally estimated at the parameter *k*.

### 5.3. Validation and Testing of the Model

In order to validate the piecewise model from Equations (39)–(41), a monotonic increasing function of stress was applied to the sensors. Monotonicity must be ensured because the hysteresis phenomenon is not embraced in this study. The following discrete stress function was applied to the sensors, but in practice, exponential, potential or linear functions could be chosen as long as they are monotonically increasing:

(47) $$\sigma_{test}[n]=G\sqrt{n}\;$$

where *n* ∈ [0,30] and *G* is equal to 56.4 KPa and 4.8 KPa for the A201-1 and the FSR 402 sensor, respectively. The value of *G* was chosen so that *σ_test_* [*n* = 30] matches for the nominal range of each device. Testing the model implies predicting sensor current based upon information from the sourcing voltage, *V_FSR_*, and the applied stress, *σ_test_*. The parameters from Table 2 and Table 3 were used to numerically solve the piecewise Equations (39)–(41). The errors associated with the proposed model and the experimental values were calculated on the basis of the Mean Absolute Error (MAE) and Root Mean Squared Error (RMSE), see Table 4.

**Figure 17:**
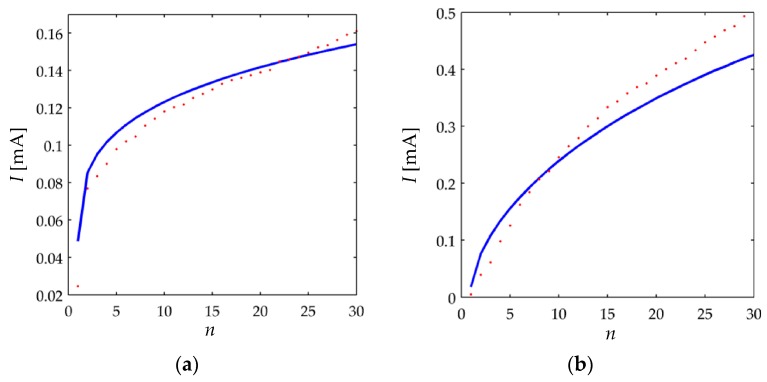
Test signal from Equation (47) exerted over the sensors: (**a**) FlexiForce A201-1 at *V_FSR_* = 3 V and (**b**) Interlink FSR 402 at *V_FSR_* = 2 V. Experimental data (red markers) and predicted current (solid blue) from Equations (39)–(41).

Figure 17 shows the experimental data for sensor current and the predicted values at different input voltages. It is clear that a better prediction is possible, but as previously stated, the Simmons’ model is approximated, and thus, the resulting parameters from the iterative process are approximated as well. 

### 5.4. Experimental Evidence Supporting a Force-Dependent Area (A) for the Current Conduction

Another contribution from this article is the proposal of a force-dependent area for current conduction. It must be recalled that the models from Zhang et al. [22], Wang et al. [23] and Kalantari et al. [9] assume *A* as a force-independent parameter. However, experimental observations based on sensor burn-out support the proposal of Equations (29) and (30).

The data fitting from Section 5.2.3 yielded parameters for the force-dependent behavior of the effective area *A*; these parameters are: *A*_0_, *A*_1_ and *A*_2_ with Equations (29) and (30) as suggested by the proposed model. If the model is evaluated at different stresses, an increasing area is expected for the incremental values of *σ*. On the other hand, when stress is constant, the application of larger *V_FSR_* increases the net current flowing through the already existing tunneling bridges in the polymer composite, but not new current paths are formed unless stress is increased; this has been discussed on Section 2.2 and on Section 5.2.3. Under such assumptions, if *V_FSR_* is sufficiently large, sensor burn-out may occur due to joule heating and the trace of current flow may be observed at different stresses.

**Figure 18:**
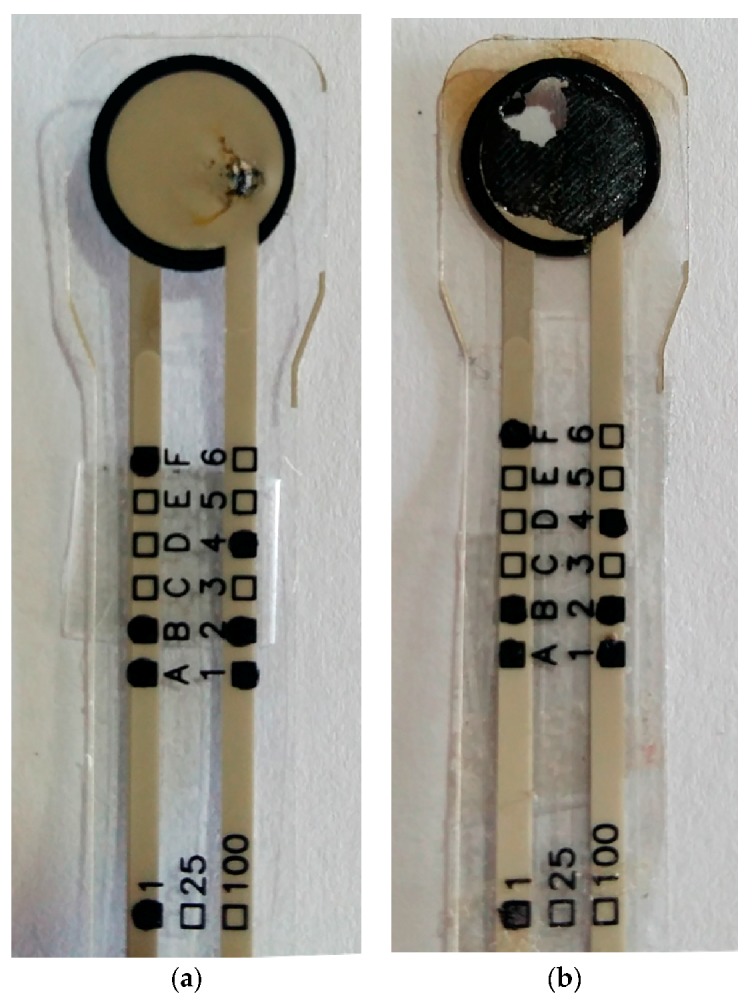
Pictures of burned-out FlexiForce sensors when loaded with (**a**) *σ* = 46 KPa at 58 V and with (**b**) *σ* = 1.8 MPa at 50 V. *A_FSR_* is equal to 41.85 mm^2^ in both cases.

In practice, different specimens of FlexiForce and Interlink sensors were tested at large stresses and large *V_FSR_*. All of them exhibited noticeable temperature increments when loaded at such conditions, but only a few FlexiForce sensors burned out. None of the Interlink sensors burned out during the experimental tests, because they exhibited a larger capability of current handling due to its comparative larger physical area, *A_FSR_*. Figure 18a shows a photo of a burned-out FlexiForce sensor when loaded at *σ =* 46 KPa at 58 V, and Figure 18b shows a picture of another specimen of FlexiForce sensor when loaded at *σ =* 1.8 MPa at 50 V. Note the small burn-out mark at Figure 18a, whereas the sensor of Figure 18b was totally scorched. The comparative behavior of the burned marks is strong evidence that the effective area *A* is force-dependent.

## 6. Conclusions and Future Work

A model for the Current-Voltage relationship (*I*-*V_FSR_*) of Force Sensing Resistors (FSRs) and polymer composites has been derived and tested. The proposed model is capable of predicting sensor current based upon information from the applied stress, σ, and sourcing voltage, *V_FSR_*. This model exhibits multiple differences compared with previous contributions to the field, as it embraces three additional parameters which have been omitted or assumed constant by previous studies. These are: the non-linear *I*-*V_FSR_* relationship, the force-dependent behavior of the effective area for current conduction (*A*), and the resistance of the conductive particles (*R_par_*) deposited in the insulating polymer; such particles behave as quantum point contacts with nonzero resistance. Experimental evidence supports the force-dependent behavior of *A*, and similarly, the nonlinear *I*-*V_FSR_* relationship has been experimentally identified by applying different voltages at constant mechanical stress.

The proposed model has been implicitly formulated by using piecewise functions. It was obtained from a combination of two concepts from quantum mechanics and one concept from classical physics. The quantum concepts are: the tunneling conduction and the resistance of quantum point contacts. The pressure dependence on the contact resistance (*R_c_*) of materials is the classical concept included in the model. 

The proposed model was tested over commercially available FSRs manufactured by Interlink Electronics, Inc and Tekscan, Inc. In general, the test results are satisfactory, especially for the contact resistance, *R_c_*. However, when modeling the tunneling conduction, a lower goodness of fit was obtained; this occurred because the literature regarding tunneling conduction was derived from a semi-classical solution for the Schrödinger equation (the WKB approximation), and due to the fact that numerical approximations were done by previous authors when deriving the *I*-*V_FSR_* relationship of thin insulating layers. Thus, one pending task is to derive a more accurate model for the current conduction of thin insulating film layers operating on the basis of quantum tunneling.

The authors discourage Wheatstone bridges, voltage dividers and multimeters as the experimental setup for reading the resistance of polymer composites (*R_bulk_*) and FSRs (*R_FSR_*). Theoretical models and experimental evidence have been presented in this study in regard to the voltage-dependent behavior of *R_bulk_* and *R_FSR_*. Wheatstone bridges, voltage dividers and multimeters operate on the basis of changing the voltage across the unknown resistance, and thus, a modulation effect is created as the applied stress and the applied voltage change simultaneously. Instead, authors encourage the usage of an amplifier in inverting configuration as the method to collect sensor data. Similarly, the voltage applied to the FSRs during testing, *V_FSR_*, should be specified by the authors in order to ensure the repeatability of results.

Future work possibilities are vast within the manufacturing and modeling of FSRs. From the manufacturing scope, the fabrication of FSRs using different materials and electrode configuration are research possibilities which are currently being explored by several authors. From the modeling standpoint, the inclusion of rheological models in the proposed model is currently a research focus of the authors; by doing this, creep compensation could be performed thus enhancing sensor performance. Similarly, the modeling and compensation of hysteresis are also being considered by the authors.

## Figures and Tables

**Table 1:** Summary of the symbols employed in the proposed model. Comparison with similar studies is also presented.

Description	Symbol	Equivalent Symbol from Zhang et al. [22]	Equivalent Symbol from Wang et al. [23]	Equivalent Symbol from Kalantari et al. [9]
Resistance of the FSR *^1^	*R_FSR_*	*R*(*σ*) *^2^	*R_r_*(*σ*) *^2^	R_total_ *^2^
Contact resistance	*R_c_*	-	-	*R_Con_*
Bulk resistance *^3^	*R_bulk_*	*R*(*σ*) *^2^	*R_r_*(*σ*) *^2^	*R_Pol_*
Voltage drop over *R_FSR_*	*V_FSR_*	*U*	*U*	*U*
Voltage drop over *R_c_*	*V_Rc_*	-	-	-
Voltage drop over *R_bulk_*	*V_bulk_*	-	-	-
Mechanical stress	*σ*	*idem*	*idem*	*idem*
Effective area at rest state *^4^	*A*_0_	- *^5^	- *^6^	- *^5^
Effective area for tunneling conduction	*A*(*σ*)	- *^5^	- *^6^	- *^5^
Power law for *A*(*σ*)	$A_{1}\sigma^{A_{2}}$	- *^5^	*N*(*σ*)*/N*(*0*) *^6^	- *^5^
Sensor physical area	*A_FSR_*	-	-	-
Contact resistance at rest state *^4^	$R_{c}^{0}$	-	-	- *^7^
Resistance of the conductive particles	*R_par_*	*idem* *^8^	*idem* *^8^	*idem* *^8^
Compressive modulus of the Polymer composite	*M*	*idem*	*idem*	*idem*
Height of the rectangular potential barrier	*V_a_*	*idem*	*idem*	*idem*
Width of the rectangular barrier at rest state *^4^	*s*_0_	*idem*	*idem*	*idem*

Hyphen symbol implies that such parameter is not embraced by the author. *^1^ Also known as the total resistance; it comprises the series connection between *R_c_* and *R_bulk_*; *^2^ Zhang et al. [22], Wang et al. [23] and Kalantari et al. [9] model the relative variation of resistance in regard to the experimentally measured resistance at rest state; see Equations (17), (23) and (24); *^3^ Also known as the tunneling resistance; *^4^ Rest state implies *σ* = 0; *^5^ The final formulations from Zhang et al. [22] and Kalantari et al. [9] assume the effective area for tunneling conduction as stress-independent , see Equations (17) and (24); *^6^ The quotient *N*(*σ*)/*N*(0) models the variation in the effective number of conductive paths with the applied stress, see Equation (23); *^7^ Although not explicitly stated by Kalantari et al. [9], the contact resistance is infinite at rest state as predicted by Equation (11); *^8^ All previous models embrace the contribution from the particles’ resistance, see Equation (12). However, they stated that *R_par_* is negligible when compared to the tunneling resistance. Hence, they discarded it as in Equation (13).

**Table 2:** Parameters obtained for the contact resistance, *R_c_*, on the basis of the proposed model of Equation (34). Parameters obtained at the third iteration.

Parameter	FlexiForce A201-1	Interlink FSR 402
*R_par_* (Ω)	2.27 × 10^−14^	394
$R_{c}^{0}$ (N·Pa^k^)	1.19 × 10^6^	1.35 × 10^10^
k *^1^	0.45	1.74
*r*^2^ *^2^	0.98	0.99

*^1^ Dimension-less parameter; *^2^ Coefficient of determination stemmed from the data fitting process.

**Table 3:** Parameters obtained from the surface fit for the piecewise Equations (39)–(41).

Parameter	FlexiForce A201-1	Interlink FSR 402
*s*_0_ (nm)	4.41	4.38
*M* (MPa)	4.73	0.388
*V_a_* (eV)	0.229	0.231
*V*th (V)	73 × 10^−3^	140 × 10^−3^
*A*_0_ (nm^2^)	3.87	145.8
*A*_1_ (nm^2^/Pa^A2^)	0.703	4.7 × 10^−6^
*A*_2_ *^1^	0.44	1.88
*r*^2^ *^2^	0.88	0.95

*^1^ Dimension-less parameter; *^2^ Coefficient of determination stemmed from the data fitting process.

**Table 4:** Mean Absolute Error (MAE) and Root Mean Squared Error (RMSE) stemmed from model testing on the basis of Equation (47).

Metric	FlexiForce A201-1	Interlink FSR 402
MAE	5.6 × 10^−6^	31.9 × 10^−6^
RMSE	7.5 × 10^−6^	37 × 10^−6^

All errors in ampere units.

**Figure A1:**
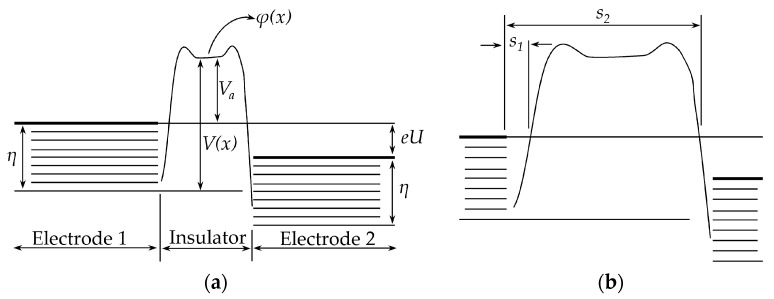
Band diagram of a thin insulating film sandwiched between to metal electrodes. (**a**) Description of the band diagram depicting electrical variables; (**b**) Zoom-in around the insulator depicting the width of the potential barrier.

## Supplementary Materials

The following are available online at http://www.mdpi.com/1424-8220/17/9/2108/s1.

## Appendix A. A Review on the Concepts of Current Density, Electrical Current and Non-Linear Voltage-Current Relationship

The current density, *J*, is defined as the electric current, *I*, per unit area of cross-section area, *A*. It can be related with the electric current as next:

(A1) I=∮J⋅dA

If the current density is constant along the cross-section area, the integral of Equation (A1) can be stated as:

(A2) I=JA

In this article, the cross-section area *A* is the effective area for the electrons to cross the potential barrier, the tunneling conduction area, and so, it must not be confused with the sensor physical dimension. In practice, *A* is in the range of square nanometers, whereas the sensor area (*A_FSR_*) is typically in the range of square millimeters. Such a great difference can be understood by recalling the fact that the electric current flows only when a conductive path is stablished between the sensor electrodes, see Figure 3b, this requires that all nanoparticles along the path get close enough to allow quantum tunneling. If a single particle is too far from another one to prevent quantum tunneling, then such current path is not formed. The net effect, is that even for a heavily loaded specimen, not all nanoparticles are conducting current simultaneously. More description on this phenomenon is available at [7,9].

The electrical resistance of a material is defined by the Ohm’s law as the relationship between the applied voltage, *U*, and the electrical current, *I*, as next:

(A3) R=U/I

However, a close form for the electrical resistance of a material cannot be always formulated, because *U* and *I* are sometimes inter-dependent. This is typical in microelectronics devices such as diodes and transistors, and this is also observed in materials working on the basis of quantum tunneling. In this article, and for the sake of full understanding, a plot is presented when the electrical resistance of a polymer composite is described; see Figure 4b and Figure 12. In general, the resistance of polymer composites show a decrease when the applied voltage increases; this is theoretically predicted by the Simmons’ model [21], and supported by experimental results from Fisher and Giaever [20].

Finally, it is common to find in literature the relationship between the applied voltage and current density.

(A4) $$R_{A}=U/J$$

The magnitude *R_A_* can be understood as the electrical resistance per unit area *A*. Equation (A4) is especially useful when theoretically describing the variations of resistance for a non-specified sensor area.

## Appendix B. General Considerations for the Derivation of the Current-Voltage (*I-U*) Relationship of Thin Insulating Film Layers

In this appendix the most important facts from Simmons´ work [21] are addressed. To authors’ criteria, Simmons made some non-obvious assumptions which are highlighted in this appendix. The practical implications of the approximations made by Simmons are also discussed.

Figure A1a shows the band diagram of a thin insulating film sandwiched between two metal electrodes. In conditions of thermal equilibrium, the top energy gap of the insulator is located above the Fermi level (*η*) of the metal electrodes. Likewise, the Fermi level of each electrode is relatively displaced from each other at a distance equal to *eU*, where *U* is the magnitude of the applied voltage across the insulating layer, i.e., *η* and *eU* are both energy quantities measured in units of electron volts.

The width of the insulating barrier is *s*, with *s = s*_2_ − *s*_1_ as shown in Figure A1b, and the height of the barrier *ϕ*(*x*) is a distance-dependent quantity with an average value given by:

(A5) $$\bar{\phi (x)}=V_{a}$$

The function *ϕ*(*x*) is the relative height of the potential barrier in regard with the Fermi level, *η*, of the metal electrodes. However, it is also possible to write the height of the potential barrier as next:

(A6) V(x)=η+ϕ(x)

Note from Figure A1a that a generic *ϕ*(*x*) has been depicted for the insulating film layer. In this appendix, only the simplest case of a rectangular potential barrier is considered, with barrier height equal to *V_a_* and barrier width equal to *s.* Nonetheless, the more general case of an image potential has been also addressed by Simmons [21].

The current flow across the thin insulating film is produced by the quantum tunneling effect. The probability (*D*(*E_x_*)) for an electron to cross the barrier, with height *V*(*x*), can be found on the basis of the WKB approximation as next:

(A7) $$D(E_{x})=\exp\left\{-\frac{4\pi }{h}\int_{s_{1}}^{s_{2}}\sqrt{2m(V(x)-E_{x})}dx\right\}$$

where *E_x_* is the kinetic energy of the electron along the *x* direction. The net current flow can be found by superposition of the electrons flowing from electrode 1 to electrode 2 minus the electrons flowing in the opposite direction. This requires computing two integrals: first, the probability for the electrons to cross the barrier as in Equation (A7), and second, the probability that the electrons have sufficient energy *E_x_*; this second integral can be found from the Fermi-Dirac distribution, *f*(*E_x_*):

(A8) $$f(E_{x})=\frac{1}{\exp\left[(E_{x}-E_{f})/kT\right]+1}$$

where *k* is the Boltzmann’s constant, *T* is the absolute temperature in Kelvin degrees and *E_f_* is the Fermi energy of the metal used to assemble the electrodes from Figure A1. However, it must be recalled from Section 2 that an FSR has multiple paths for particle transmission, and the role of the conductive nanoparticles is to allow electron bridging between consecutive barriers, see Figure 3. Hence, an FSR has actually two different fermi levels: one Fermi level for the metal electrodes and the other one for the conductive nanoparticles. This fact has not been considered by Simmons and may turn irrelevant in practice, but it is important to recall that the Simmons’ derivation assumed a single Fermi level.

The above mentioned integrals are scaled by the number of free electrons per unit volume, i.e., the number of electrons in the conduction band per unit volume. This calculation is not here presented but it can be found in detail at Equations (2)–(7) in Simmons’ work [21]. Such calculation required a coordinate change, so that *E_x_* is expressed in polar coordinates, *E_r_*.

Finally, the net current density can be found from superposition as next:

(A9) $$J=\int_{0}^{Em}D(E_{x})\zeta dE_{x}$$

where ζ = ζ_1_ − ζ_2_ and

(A10) $$\zeta_{1}=\frac{4e\pi m^{2}}{h^{3}}\int_{0}^{\infty }f(E)dE_{r}$$

(A11) $$\zeta_{2}=\frac{4e\pi m^{2}}{h^{3}}\int_{0}^{\infty }f(E+eU)dE_{r}$$

Caution is necessary when reading the original formulation of Equation (A11), that is, the Equation (7) at [21], because the external applied voltage is labeled as *V* by Simmons, but previously, the same letter has been used by him for the height of the potential barrier, as in Equation (A6). For this reason, the external applied voltage is labeled as *U* in this appendix.

The calculation of the integrals is not here addressed, but it can be found in detail at Equations (9)–(19) in [21]. The final expression is next presented:

(A12) $$J=J_{0}\left\{V_{a}\exp\left[-BV_{a}^{1/2}\right]-(V_{a}+eU)exp\left[-B{\left(V_{a}+eU\right)}^{1/2}\right]\right\}$$

where

$$J_{0}=e/2\pi h{\left(\beta s\right)}^{2}$$

and

$$B=(4\pi \beta s/h){\left(2m\right)}^{1/2}$$

The term *β* is described ahead, but first note that Equation (A12) is the sum of two exponential terms biased by the external voltage, *U*; this is so because of the superposition principle that predicts electrons flowing from electrode 1 to electrode 2 and vice versa.

Simmons calculated the correction factor *β* on each interval of *U* in regard with *V_a_/e*. Given an arbitrary function *f*(*x*), the value of *β* can be taken as the maximum error committed when the following approximation is done:

(A13) $$\int_{s_{1}}^{s_{2}}f^{1/2}(x)dx\approx f^{1/2}(s_{2}-s_{1})$$

Note that the approximation from Equation (A13) is especially useful when evaluating the integral from Equation (A7). In practice, the maximum error stemmed from (A13) depends upon the external applied voltage, *U*. As previously mentioned on Section 2, the maximum error stemmed from (A13) is equal to 6% when *U* < *V_a_*/*e*. No error is yielded when *U ≈ 0* as in Equation (6).

The correction factor *β* is a scalar quantity that compensates the error on each of the aforesaid intervals. Unfortunately, when *U* < *V_a_*/*e* the compensation factor is not a scalar, but rather a voltage-depend quantity. Simmons solved this controversy by considering *β = 1* in the intervals *U ≈ 0* and *U* < *V_a_*/*e*, and *β = 23/24* when *U* > *V_a_*/*e*.

The practical consequences of such an approximation is that a discontinuous output is yielded when switching between models at the limit voltages; this discontinuity is especially remarkable when *U* = *V_a_*/*e*; as previously shown on the plot of Figure 4a The final expressions for the *J-U* relationship in thin insulating layers have been previously presented in Section 2, see Equations (6)–(8).

Finally, the relationship between *J* and *I* has been presented in Equations (A1) and (A2), but caution is required when using them, because the area *A* is the effective area for the electrons to cross the potential barrier *ϕ*(*x*), and so, it must not be confused with the physical area of the FSR, *A_FSR_*.
